# APOL2 Stabilizes Ku80 to Confer NHEJ‐Mediated Radioresistance in Gastric Cancer

**DOI:** 10.1002/advs.202506294

**Published:** 2025-09-15

**Authors:** Dan Zu, Qimei Bao, Hanyi He, Yuke Zhong, Mingcong Deng, Yangchan Hu, Chunkai Zhang, Chen Liang, Yixing Huang, Haidong Liu, Xiao Li, Yanhua He, Guoyan Luo, Weixin Wu, Fenghui Guan, Shengfeng Xu, Min Liu, Albino Bacolla, Ji Jing, Yian Du, John A. Tainer, Yin Shi, Zu Ye, Xiangdong Cheng

**Affiliations:** ^1^ School of Life Sciences Tianjin University Tianjin 300100 China; ^2^ Zhejiang Cancer Hospital Hangzhou Institute of Medicine (HIM) Chinese Academy of Sciences Hangzhou 310022 China; ^3^ Postgraduate training base Alliance of Wenzhou Medical University (Zhejiang Cancer Hospital) Hangzhou 310022 China; ^4^ Key Laboratory of Prevention Diagnosis and Therapy of Upper Gastrointestinal Cancer of Zhejiang Province Hangzhou 310022 China; ^5^ Zhejiang Provincial Research Center for Upper Gastrointestinal Tract Cancer Zhejiang Cancer Hospital Hangzhou 310022 China; ^6^ Hangzhou Medical College Hangzhou 310013 China; ^7^ College of Pharmaceutical Science Zhejiang University of Technology Hangzhou 310014 China; ^8^ Zhejiang University School of Medicine Hangzhou 310058 China; ^9^ Human Genome Sequencing Center Baylor College of Medicine Houston TX 77030 USA; ^10^ Department of Molecular and Cellular Oncology The University of Texas MD Anderson Cancer Center Houston TX 77030 USA

**Keywords:** APOL2, gastric cancer, Ku80, NHEJ, ubiquitination

## Abstract

Radiotherapy is one of the most important adjuvant treatment methods for gastric cancer (GC). However, radioresistance remains a major clinical obstacle. In this study, APOL2 is identified as a key player in promoting non‐homologous end joining (NHEJ)‐mediated double‐strand break (DSB) repair and enhancing radioresistance in GC. Bioinformatics and clinical data revealed that high APOL2 expression is correlated with poor prognosis in GC patients. Functional experiments showed that APOL2 overexpression enhances genomic stability by accelerating DSB repair via the NHEJ pathway, while APOL2 knockout impairs repair capacity. Mechanistically, APOL2 binds to and stabilizes Ku80 by enhancing USP7‐mediated deubiquitylation, thereby increasing Ku80 protein levels to promote NHEJ repair, ultimately conferring radioresistance. Moreover, high‐throughput screening identified formononetin (FN) as a small molecule capable of disrupting the APOL2‐Ku80 interaction, thereby restoring radiosensitivity in GC cells. Our findings underscore the role of APOL2 in mediating radioresistance through Ku80 stabilization and highlight FN as a potential therapeutic agent to counteract radioresistance in GC treatment.

## Graphical abstract

Radiotherapy resistance in gastric cancer (GC) is often mediated by enhanced DNA repair. APOL2 is found to promote NHEJ by stabilizing Ku80, conferring radioresistance. High APOL2 correlates with poor prognosis. Formononetin disrupts APOL2‐Ku80, restoring radiosensitivity, suggesting a novel therapeutic strategy.

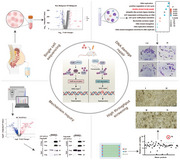

## 1 Introduction

DNA damage repair is vital for genomic stability, with double‐strand breaks (DSBs) representing one of the most lethal types of DNA damage.^[^ ^1^, ^2^ ^]^ Various internal and external factors, such as ionizing radiation (IR), ultraviolet light, and metabolic byproducts, can induce DSBs and threaten cellular integrity.^[^ ^2^, ^3^ ^]^ Nonhomologous end joining (NHEJ) is the primary and most rapid pathway for DSB repair, particularly in somatic cells, where it functions independently of the cell cycle and efficiently restores DNA integrity by directly ligating the broken DNA ends.^[^ ^4^ ^]^ Unlike homologous recombination (HR), which requires an intact DNA template and occurs mainly in the S and G2 phases of the cell cycle, NHEJ can operate at any cell cycle stage, making it indispensable for quick and flexible DNA repair.^[^ ^4^, ^5^, ^6^ ^]^ However, the lack of template dependency in NHEJ repair can lead to small insertions or deletions, which may contribute to genomic instability under certain conditions.

The role of NHEJ is particularly significant in cancer.^[^ ^7^ ^]^ While DSB repair pathways are essential for normal cell survival, tumor cells often exploit NHEJ to accumulate growth‐promoting mutations, including those caused by DSB‐inducing treatment regimens.^[^ ^8^, ^9^ ^]^ Indeed, tumor cells with efficient NHEJ can quickly repair radiation‐induced DNA breaks, thereby resisting the cytotoxic effects of radiotherapy (RT).^[^ ^10^, ^11^, ^12^ ^]^ Given the central role of NHEJ in mediating DNA repair, particularly in response to therapeutic interventions such as radiation, it is crucial to understand the specific molecular components of this pathway and their regulation in tumor cells. Insights into how tumor cells leverage NHEJ for survival could lead to targeted approaches aimed at sensitizing cancer cells to RT and overcoming resistance, which ultimately improves treatment outcomes.

Gastric cancer (GC) is one of the most common malignant tumors in the world, and its occurrence is influenced by many factors, including genetics, environment, and lifestyle.^[^ ^13^ ^]^ Patients with advanced GC are typically treated with a combination of surgery, chemotherapy, and RT.^[^ ^14^ ^]^ RT primarily targets DNA by inducing DSBs, thereby inhibiting cell proliferation and inducing apoptosis.^[^ ^15^ ^]^ The INT‐0116 clinical trial in the United States revealed that patients who received chemotherapy and radiation after surgery had better survival rates than those who received surgery alone.^[^ ^16^ ^]^ However, resistance to RT is a significant obstacle to treatment efficacy, often resulting in cancer recurrence and poor prognosis. Thus, there is an urgent need for therapeutic strategies that improve the radiosensitivity of GC cells.

Genetic factors have been identified as key contributors to the development, progression, and treatment response of GC. In particular, the aberrant expression of specific genes has been linked to radiosensitivity, tumor cell proliferation, apoptosis, and invasion.^[^ ^6^, ^17^ ^]^ We analyzed data from The Cancer Genome Atlas (TCGA) and clinical GC samples to identify potential targetable genes related to tumor stage and poor prognosis in GC patients. Our data revealed that expression of apolipoprotein L family member 2 (APOL2), which encodes a member of the phospholipid transporter family,^[^ ^18^ ^]^ was significantly increased in GC tissues and associated with poor clinical outcomes in patients with the highest expression. APOL2 modulates cell proliferation and contributes to breast and colorectal tumorigenesis.^[^ ^19^ ^]^ Aberrant APOL2 expression may inhibit apoptosis, allowing tumor cells to survive and continue to proliferate.^[^ ^20^, ^21^ ^]^ However, the role of APOL2 in GC has not yet been reported.

In this study, APOL2 expression in GC was found to be positively correlated with tumor progression, suggesting a potential association with poor prognosis. Functionally, we found that APOL2 increases the resistance of GC cells to IR, both in vitro and in vivo. APOL2 binds to and stabilizes Ku80 via USP7‐mediated deubiquitylation, resulting in Ku80 accumulation that enhances NHEJ repair efficiency and ultimately confers radioresistance. Importantly, formononetin (FN) effectively disrupts the APOL2‐Ku80 interaction and restores radiosensitivity. In summary, our study aims to elucidate the role of APOL2 in GC radioresistance and identify a potential strategy for overcoming radioresistance using FN. These findings are expected to contribute to the development of targeted therapies that improve the radiosensitivity and clinical outcomes of GC patients.

## 2 Results

### 2.1 High APOL2 Expression is Associated with Poor Prognosis in GC Patients

**Figure 1:**
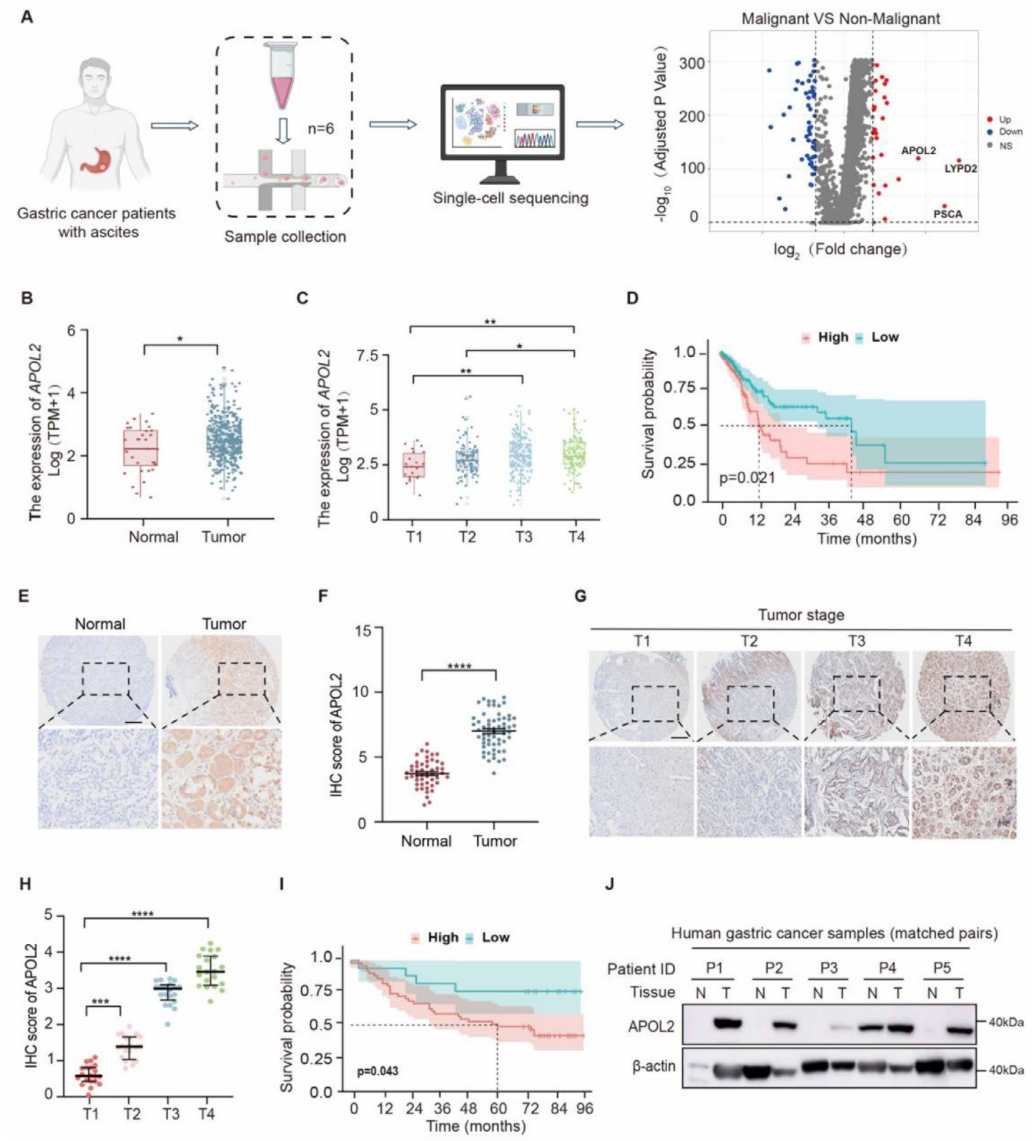
APOL2 is highly expressed in GC and is associated with poor prognosis. A) Volcano plot of RNA‐seq data in ascites single cells from GC patients, revealing strong upregulation of APOL2. B) Box plot of *APOL2* mRNA levels in normal and GC tissues from the TCGA database. C) APOL2 expression across T stages (T1–T4) in GC. D) KM curve of the correlation between APOL2 expression and OS in GC patients in the TCGA database (log‐rank test). E,F) Representative APOL2 IHC staining in normal (*n* = 48) and GC (*n* = 60) tissues (E), with corresponding scores (F). Scale bar, 50 µm. G,H) Representative images (G) and scores (H) of APOL2 IHC at different stages in normal and GC tissues. Scale bar, 50 µm. I) KM curve of the correlation between *APOL2* expression and survival in GC patients (log‐rank test). J) Western blot analysis of APOL2 protein expression in paired tumor and adjacent normal tissues from five stage III GC patients. Statistical analysis was performed via a two‐tailed unpaired Student's t‐test. Data was presented as mean ± SD; *P < 0.05, **P < 0.01, ***P < 0.001, ****p < 0.0001. All data are representative of three independent experiments.

Single‐cell RNA sequencing of malignant ascites from GC patients was previously performed by our group.^[^ ^22^ ^]^ The volcano map of differential gene expression between malignant and non‐malignant controls showed that APOL2, LYPD2, and PSCA were the most upregulated genes (**Figure** **1**A). We analyzed the data from TCGA and found that LYPD2 expression was not significantly different between tumor and normal tissues in patients with GC, and that LYPD2 expression was not correlated with clinical stage or prognosis (Figure S1A–C, Supporting Information). PSCA was expressed at lower levels in tumor tissues than in control tissues and was not associated with clinical stage or prognosis (Figure S1D–F, Supporting Information). Conversely, APOL2 exhibited markedly higher expression levels in tumor tissues compared with normal tissues (Figure 1B). Therefore, we explored the relationship between APOL2 expression and clinicopathological characteristics. The results revealed that APOL2 expression was correlated with clinical stage (Figure 1C). Kaplan‐Meier (KM) curve analysis revealed that increased APOL2 expression was associated with reduced overall survival (OS) (Figure 1D), which led us to further explore the association between APOL2 expression and tumor growth. To validate these findings, we collected 108 clinical samples from GC patients to evaluate the levels of APOL2 protein in tumor tissues and their corresponding adjacent normal tissues via immunohistochemical staining. Our findings corroborated those of previous analysis by demonstrating that APOL2 was markedly higher in GC tissues than in normal tissues and was significantly correlated with clinical stage (Figure 1E–H). Furthermore, analysis of patient prognosis revealed that increased APOL2 protein levels were associated with poor clinical outcomes, which was consistent with the TCGA results (Figure 1I). To further verify these observations, we performed Western blot analyses on five paired samples of tumor and adjacent normal tissues, which confirmed that APOL2 expression was significantly higher in tumor tissues compared to adjacent normal tissues (Figure 1J). These results show that high APOL2 gene expression leads to increased protein levels and is a biomarker for robust tumor progression and poor prognosis in GC patients.

### 2.2 APOL2 Enhances DNA Damage Response and Promotes Genomic Stability

**Figure 2:**
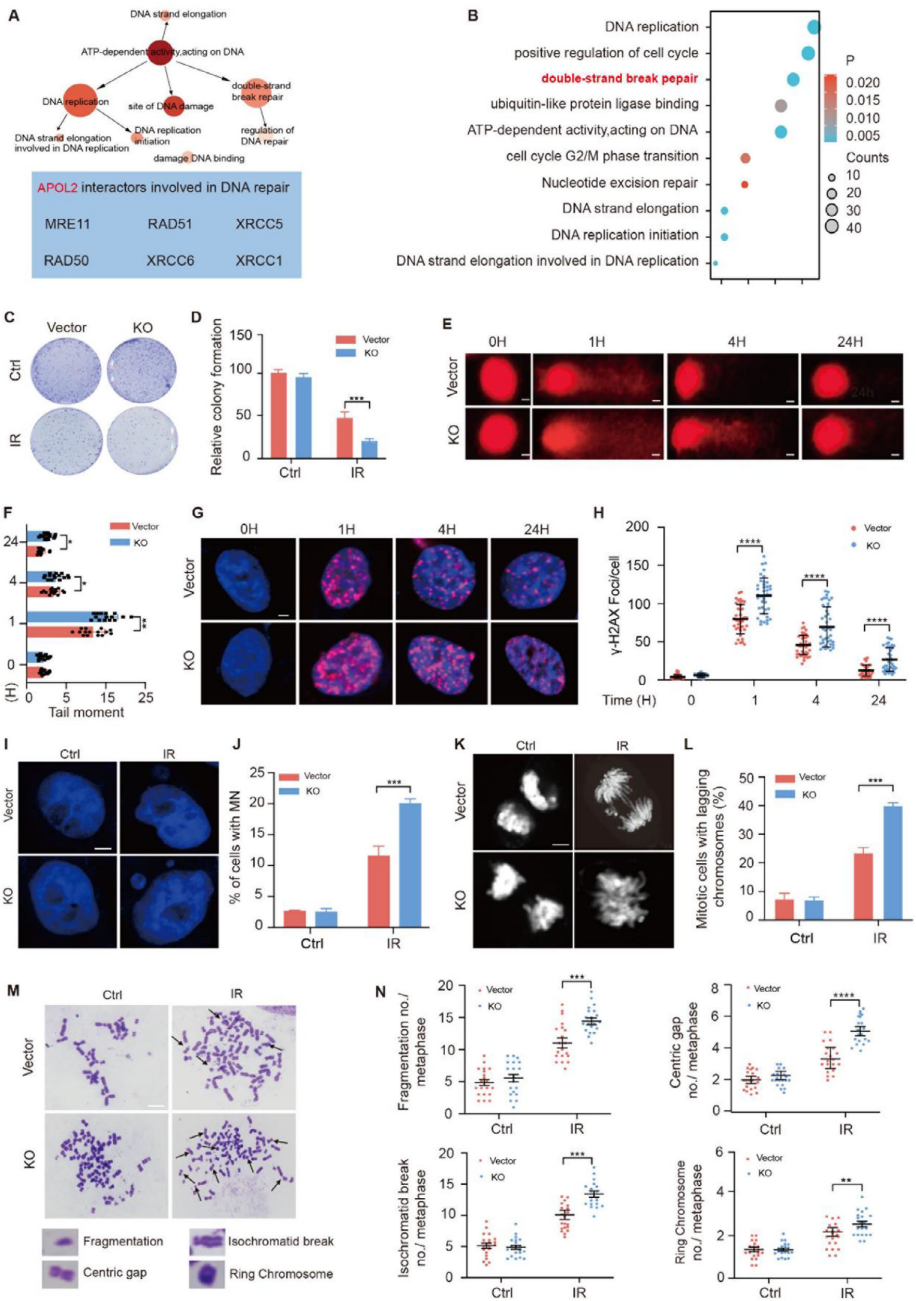
APOL2 enhances DNA damage response and promotes genomic stability. A) *APOL2‐*associated DDR nucleoproteins grouped according to known functions. The table shows key proteins involved in DNA damage repair. B) KEGG enrichment analysis of NC cells and APOL2‐OE cells showed significantly different signaling pathways. C,D) Representative images and corresponding survival score curves of the colony formation assay in the vector and APOL2‐KO groups at a radiation dose of 3 Gy (*n* = 3). E,F) Representative comet assay images (E) and tail moment quantification (F) uesd to assess DSB repair at the indicated time points following 3 Gy IR exposure (*n* = 15). Scale bar, 10 µm. G,H) Representative immunofluorescenc images (G) and quantitative analysis (H) of γ‐H2AX foci changes at 1, 4, and 24 h after exposure to 3 Gy irradiation dose (*n* = 40). Scale bar, 10 µm. I,J) Representative images I) of micronucleus formation in APOL2‐KO cells treated with 3 Gy IR after 24 h and quantitative analysis of the percentage of micronucleated cells (MNs) relative to the total number of cells (J). DAPI staining showed nuclei (*n* = 20). Scale bar, 25 µm. K,L) Quantitative analysis of representative chromosome images (K) and the percentage of APOL2‐KO cells with chromosome lagging (L) 24 h after 3 Gy IR treatment (*n* = 20). Scale bar, 25 µm. M,N) Representative images (M) and quantitative analysis (N) of chromosomal aberrations in APOL2‐KO cells treated with 3 Gy IR after 24 h (*n* = 15). Scale bar, 25 µm. Statistical analysis was performed via two‐way ANOVA. Data was presented as mean ± SD; ^*^ *P* < 0.05, ^**^ *P* < 0.01, ^***^ *P* < 0.001, ^****^ *P* < 0.0001. All data are representative of three independent experiments.

To further characterize the role of APOL2 in GC, we measured the expression level of APOL2 in different GC cell lines. APOL2 exhibited the lowest expression in MKN1 cells but the highest expression in HGC27 cells (Figure S2A, Supporting Information). Therefore, we established APOL2 stably overexpressing cell lines (APOL2‐OE) in MKN1 cells and generated APOL2‐knockout cell lines (APOL2‐KO) in HGC27 cells (Figure S2B, Supporting Information). To elucidate the molecular function of APOL2, we performed co‐immunoprecipitation (Co‐IP) of APOL2 followed by liquid chromatography‐mass spectrometry (LC‐MS) analysis. By analyzing nuclear interactions, we identified multiple DDR proteins as binding partners of APOL2 (**Figure** **2**A **;** Figure S2C,D, Supporting Information). Kyoto Encyclopedia of Genes and Genomes (KEGG) pathway analysis further suggested an association between APOL2 and DNA damage repair signaling (Figure 2B). Thus, we proceeded to investigate the potential role of APOL2 in DNA damage repair, particularly in modulating radiosensitivity in GC.

Following exposure to varying doses of IR and different post‐irradiation recovery periods, APOL2‐OE cells exhibited significantly enhanced radioresistance compared with control cells (NC), with the most marked difference observed at 3 Gy, while higher radiation doses ultimately led to substantial cell death (Figure S3A–D, Supporting Information). In addition, compared with control cells, APOL2‐OE cells exhibited significantly less γ‐H2AX foci formation (a well‐established marker of DSBs) and less micronuclei formation (a well‐known indicator of genomic instability) post‐irradiation (Figure S3E,F, Supporting Information). Notably, quantitative analysis revealed that the radiation‐induced differences between experimental groups reached saturation at 3 Gy irradiation across all assays. We therefore used this dose for subsequent experiments. We further evaluated the function of APOL2 in radiation‐induced DNA damage response in GC cells by colony formation assay and found that APOL2 knockout significantly attenuated post‐irradiation clonogenic survival, while its overexpression conferred significant radioprotection, as evidenced by enhanced colony formation. These results demonstrate APOL2's role in DNA damage repair (DDR) pathways (Figure 2C,D; Figure S4A, Supporting Information). Comet assay analysis demonstrated that APOL2 knockout significantly increased the tail moment, while APOL2 overexpression reduced this DNA damage parameter, indicating APOL2 promotes post‐radiation DNA repair (Figure 2E,F; Figure S4B, Supporting Information). As IR induces DSBs in DNA and triggers γ‐H2AX foci formation at DNA damage sites, we assessed the effect of APOL2 on γ‐H2AX foci formation in IR‐induced GC cells through immunofluorescence staining. Immunofluorescence analysis revealed that APOL2 knockout significantly increased γ‐H2AX foci formation post‐irradiation, whereas APOL2 overexpression reduced foci numbers, suggesting its potential involvement in promoting DNA DSB repair (Figure 2G,H; Figure S4C, Supporting Information).

Notably, defective DNA damage repair induces genomic instability, triggering micronucleus formation and chromosomal aberrations.^[^ ^22^ ^]^ Therefore, we next explored whether APOL2 affects the genomic stability of GC cells. Micronucleus assays demonstrated that APOL2 knockout exacerbated radiation‐induced micronuclei formation, while its overexpression attenuated this genomic instability marker (Figure 2I,J; Figure S4D, Supporting Information). These results underscore the role of APOL2 in promoting post‐irradiation DNA repair and maintaining genomic stability. Likewise, the results of chromosome lag experiments, which monitor chromosome segregation at anaphase, revealed that APOL2 knockout significantly increased the frequency of cells with lagging chromosomes during anaphase, while its overexpression reduced this mitotic aberration, suggesting APOL2 plays a role in maintaining post‐irradiation chromosomal segregation fidelity (Figure 2K,L; Figure S4E, Supporting Information). Moreover, chromosomal aberration assays also demonstrated that APOL2 knockout significantly increased the frequency of aberrant chromosomes, including fragmentation, isochromatid breaks, isochromatid gaps, and centric gaps, while its overexpression markedly reduced these abnormalities, indicating APOL2's crucial role in maintaining genomic stability (Figure 2M,N; Figure S4F, Supporting Information). As highly cytotoxic lesions, unrepaired or misrepaired DSBs could trigger cell lethality. Flow cytometric analysis revealed that APOL2 knockout sensitized cells to radiation‐induced apoptosis, while its overexpression conferred significant protection against radiation‐mediated cell death, indicating its radioprotective function (Figure S5A,B, Supporting Information).

Collectively, these results establish APOL2 as a key regulator of radioprotection by promoting IR‐induced DNA damage repair, maintaining genomic integrity, and ultimately conferring radioresistance in GC cells.

### 2.3 APOL2 Facilitates NHEJ‐Mediated DSB Repair

**Figure 3:**
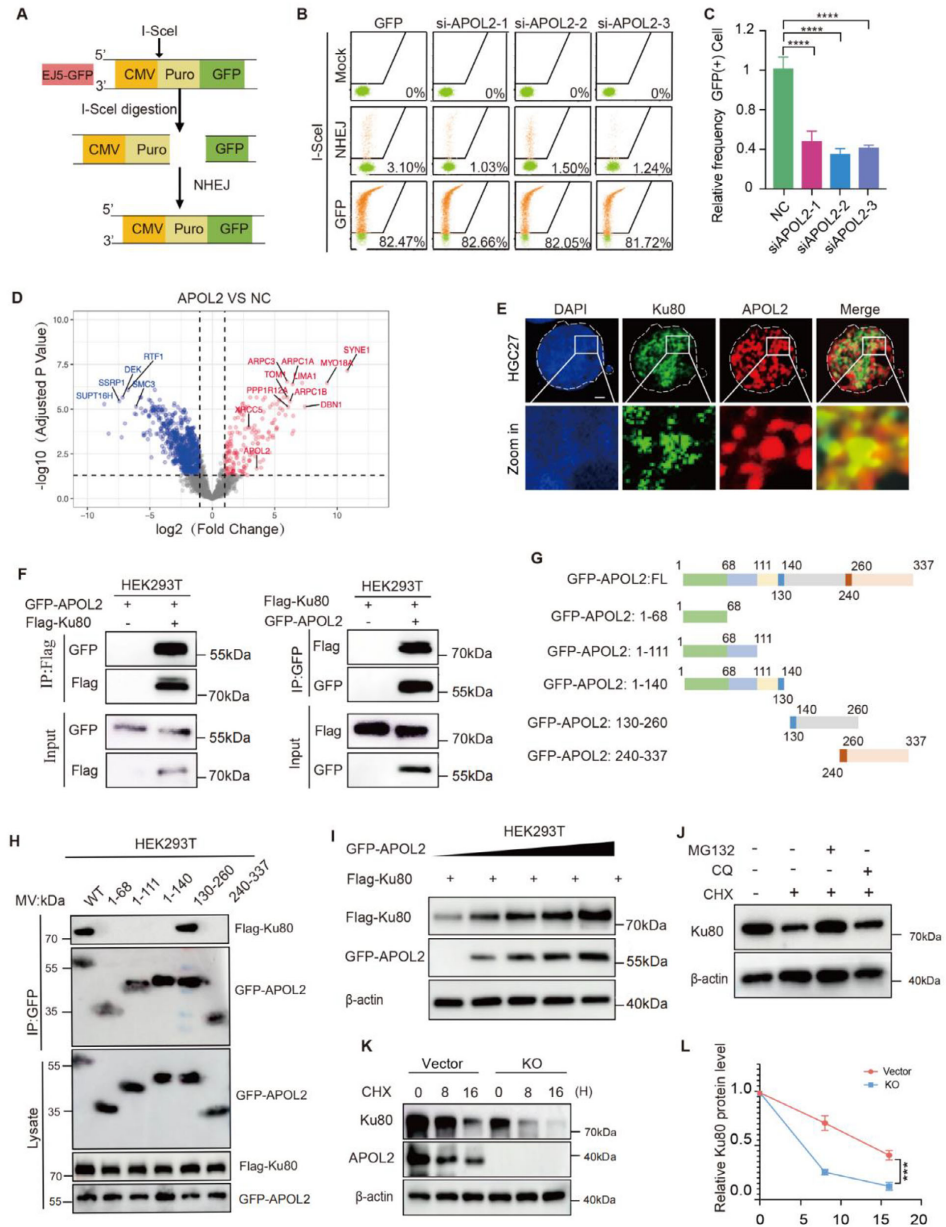
APOL2 binds to and stabilizes Ku80. A) Schematic diagram of plasmid construction to assess NHEJ activity. B‐C) Representative flow cytometry images (B) and quantitative analysis of relative GFP‐positive cell percentages (C) for I‐SceI‐infected and uninfected cells treated with the hEJ5‐GFP reporter system (*n* = 3). D) Volcano plot showing significant differential expression patterns in NC versus APOL2‐OE (upregulated genes are in red; downregulated genes are in blue (|log2FC | ≥ 1 and q‐value ≤ 0.05). E) Representative immunofluorescence images demonstrating the colocalization of APOL2 (red) and Ku80 (green) upon IR. Nuclear DNA was counterstained with DAPI (blue). Scale bar, 10µm. F) Co‐IP experiments revealed the exogenous interaction between APOL2 and Ku80 in HEK293T cells. G) Diagram of plasmid constructs encoding full‐length (WT) and truncated APOL2 mutants. H) IP and Western blot analysis of HEK293T lysates from cells collected 48 h after transfection with the indicated expression vector and probed with the indicated antibodies. I) The protein expression levels of Ku80 and APOL2 in HEK293T cells transfected with Flag‐Ku80 and GFP‐APOL2 or empty vector plasmids were measured via Western blot assays. J) Protein expression levels of Ku80 in HEK293T cells after treatment with 50 µm CQ or 10 µm MG132. K‐L). The effect of CHX (100 µg mL^−1^) treatment in HGC27 cells stably transfected with APOL2 KO and vector plasmids (K), as well as in MKN1 cells stably transfected with GFP‐APOL2 and NC plasmids (L) (*n* = 3). Statistical analysis was performed via two‐tailed unpaired Student's t‐tests (C) or two‐way ANOVA (L). Data was presented as mean ± SD; ^***^ *P* < 0.001, ^****^ *P* < 0.0001. All data are representative of three independent experiments.

HR, NHEJ, microhomology‐mediated end joining (MMEJ), and single‐strand annealing (SSA) are the main DNA repair mechanisms of eukaryotic cells in response to IR‐induced DSBs.^[^ ^1^ ^]^ To identify the specific DSB repair pathway involving APOL2, we quantified the HR, NHEJ, MMEJ, and SSA activities using DR‐GFP, EJ5‐GFP, EJ2‐GFP, and SA‐GFP reporter assays, respectively (**Figure** **3**A; Figure S6, Supporting Information). Under equivalent transfection efficiency conditions, APOL2 knockdown significantly impaired NHEJ repair activity, while the repair efficiencies of HR, SSA, and MMEJ remained unaltered, demonstrating that APOL2 specifically regulates NHEJ‐mediated DSB repair (Figure 3B,C; Figure S6, Supporting Information).

To further understand how APOL2 regulates NHEJ repair, we examined the effect of APOL2 on key NHEJ pathway components. NHEJ begins with the recognition and binding of Ku70/Ku80 heterodimers to the ends of DNA breaks, followed by DNA‐PKcs recruitment to form DNA‐PK holoenzyme complexes.^[^ ^23^ ^]^ Our results showed that after IR‐induced DNA damage, APOL2 deletion significantly impaired IR‐induced Ku70/Ku80 foci formation, whereas APOL2 overexpression significantly increased the foci number of Ku80 and Ku70 in GC cells, suggesting that APOL2 facilitates early NHEJ pathway activation (Figure S7A–D). As the central regulatory kinase in NHEJ, DNA‐PKcs could be recruited to the DSBs to form heterodimers with Ku70/Ku80, where autophosphorylation induces its full activation and initiates the repair cascade.^[^ ^24^ ^]^ Intriguingly, upon IR‐induced DNA damage, APOL2 did not affect the expression of DNA‐PKcs, suggesting that its regulatory role occurs independently of DNA‐PKcs expression modulation (Figure S7E,F, Supporting Information). However, APOL2 knockout attenuated DNA‐PKcs phosphorylation in GC cells, while its overexpression enhanced DNA‐PKcs phosphorylation, demonstrating APOL2‐dependent regulation of DNA‐PKcs activation (Figure S7G,H, Supporting Information).

In conclusion, our findings demonstrate that APOL2 orchestrates IR‐induced DSB repair by facilitating the NHEJ pathway.

### 2.4 APOL2 Binds to and Stabilizes Ku80

To further elucidate the molecular mechanism by which APOL2 facilitates NHEJ‐mediated DSB repair, we performed quantitative proteomic analysis of APOL2 interactome, which identified Ku80 (XRCC5) as a significantly enriched binding partner of APOL2 (Figure 3D). Given the pivotal role of Ku80 in NHEJ repair,^[^ ^4^ ^]^ we prioritized it for further validation based on our earlier findings that APOL2 promotes NHEJ. Immunofluorescence analysis in GC cells revealed robust nuclear co‐localization between endogenous APOL2 and Ku80 (Figure 3E; Figure S8A, Supporting Information), suggesting spatial proximity for functional cooperation. To biochemically validate this interaction, we performed Co‐IP assays in both exogenous and endogenous systems and confirmed the direct interactions of APOL2 and Ku80 under physiological conditions in GC cells (Figure 3F; Figure S8B, Supporting Information). Generally, Ku80 and Ku70 form a complex to function.^[^ ^25^ ^]^ It can identify DSBs and initiate the NHEJ process.^[^ ^26^ ^]^ We confirmed the interaction between APOL2 and Ku70 through Co‐IP (Figure S8C,D, Supporting Information). However, in vitro GST pull‐down experiments using purified proteins indicated that APOL2 only binds to Ku80, but not to Ku70 (Figure S8E, Supporting Information). To investigate which APOL2 domain mediates the interaction with Ku80, we constructed a series of APOL2 truncations, which showed that aa 130–260 likely contains a prominent domain involved in the APOL2‐Ku80 interaction (Figure 3G,H).

To investigate the pathological association between APOL2 and Ku80 and evaluate its clinical implications, we analyzed the clinical proteomics data of GC patient samples collected from Zhejiang Cancer Hospital, which revealed a significant positive correlation between APOL2 and Ku80 protein levels (Figure S8F, Supporting Information). Notably, TCGA database analysis showed no such correlation at the mRNA level, implying post‐translational regulation of Ku80 by APOL2 (Figure S8G, Supporting Information). We then divided the GC patients into different groups according to APOL2 expression and Ku80 expression. Strikingly, KM survival analysis demonstrated that patients with concurrent high APOL2 and Ku80 expression had the poorest prognosis, while neither low APOL2 expression and low Ku80 expression nor high APOL2 expression and low Ku80 expression groups showed significant survival differences from the median cohort survival (Figure S8H, Supporting Information). These findings indicate that the prognostic impact of APOL2 is strictly dependent on Ku80.

To further confirm the correlation between APOL2 and Ku80 protein expression, we performed the experiments in GC cell lines and found that APOL2 overexpression increased Ku80 protein levels, while neither APOL2 overexpression nor APOL2 knockout altered Ku80 mRNA levels (Figure 3I; Figure S8I,J, Supporting Information). Hence, we speculate that this regulation occurred post‐transcriptionally. To determine the degradation pathway(s) involved, we treated cells with chloroquine (CQ, an autophagy inhibitor) or MG132 (a proteasome inhibitor). Strikingly, only the proteasome inhibitor MG132 rescued Ku80 protein levels (Figure 3L), unequivocally demonstrating proteasome‐dependent Ku80 degradation. To directly assess the role of APOL2 role in modulating Ku80 degradation, we performed cycloheximide (CHX, 100 µg/mL) chase assays in both gain and loss‐of‐function models. Our results indicated that APOL2 knockout accelerated Ku80 degradation, whereas APOL2 overexpression prolonged the Ku80 half‐life (Figure 3K,L; Figure S8K,L, Supporting Information). These complementary approaches consistently demonstrated that APOL2 specifically stabilizes Ku80 protein by attenuating its proteasomal degradation.

Collectively, our study demonstrates that APOL2 directly binds to and stabilizes Ku80, implying that the APOL2‐Ku80 axis may constitute a functionally significant and clinically relevant mechanism governing NHEJ repair in GC.

### 2.5 APOL2 Stabilizes Ku80 via USP7‐Mediated Deubiquitination

**Figure 4:**
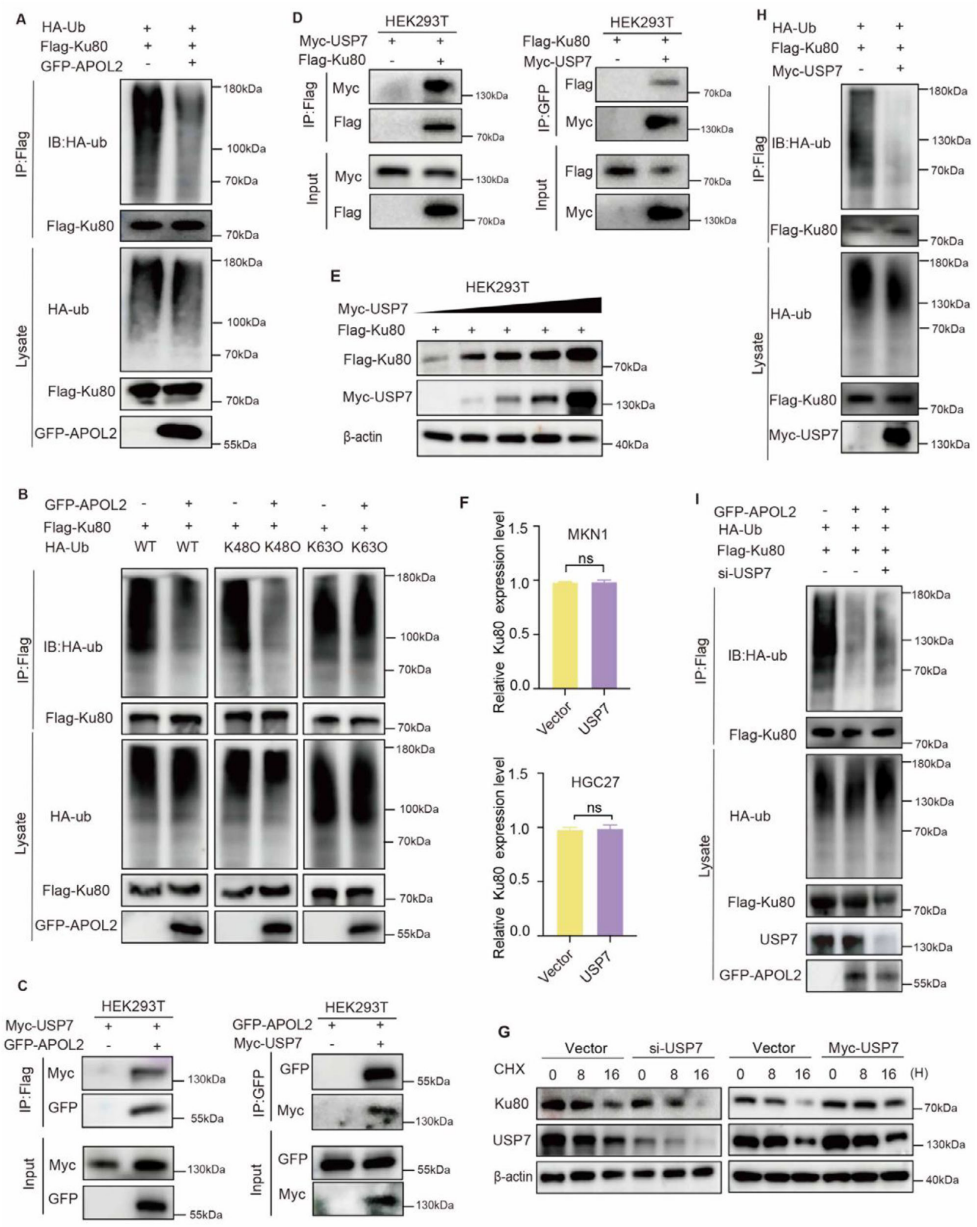
APOL2 stabilizes Ku80 via USP7‐mediated deubiquitination. A) Ubiquitination assay of Ku80 in HEK293T cells co‐transfected with Flag‐Ku80, HA‐Ub, GFP‐APOL2, or empty vector plasmids following treatment with 10 µm MG132 for 6 h. Total cell lysates were immunoprecipitated with anti‐Flag, followed by Western blot to detect ubiquitinated Ku80. B) Ubiquitination assay of Ku80 in HEK293T cells co‐transfected with HA‐Ub, HA‐Ub (complete Lys48 residue only), HA‐Ub (complete Lys63 residue only), GFP‐APOL2, or empty vector plasmids. Following 10 µm MG132 treatment for 6 h, immunoprecipitation (IP) was performed using anti‐Flag antibody with subsequent Western blot analysis using ubiquitination antibody. C) Co‐IP assays in HEK293T cells co‐transfected with GFP‐APOL2 and Myc‐USP7 revealed exogenous interactions between APOL2 and Myc‐USP7. D) Co‐IP assays in HEK293T cells transfected with Myc‐USP7 and Flag‐Ku80 reveal exogenous interactions between Myc‐USP7 and Ku80. E) Ku80 protein expression levels in HEK293T cells transfected with Flag‐Ku80 and increasing concentrations of Myc‐USP7 (0–5 µg) or empty vector plasmids were detected by Western blot. F) The expression level of Ku80 mRNA in MKN1 or HGC27 cells co‐transfected with control or Myc‐USP7 overexpression plasmids was quantified by qRT‐PCR (*n* = 3). G) Assessment of Ku80 protein stability using CHX chase assay. HGC27 cells stably expressing GFP‐APOL2 or empty vector (negative control) were treated with CHX (100 µg mL^−1^) for 0, 8, and 16 h. H) Ku80 ubiquitination was analyzed in HEK293T cells co‐transfected with Flag‐Ku80, HA‐Ub, and Myc‐USP7 or empty vector plasmids following treatment with 10 µm MG132 for 6 h. I) Ubiquitination assay of Ku80 in HEK293T cells co‐transfected with GFP‐APOL2, si‐USP7, Flag‐Ku80, HA‐Ub, or empty vector plasmids and treated with 10 µm MG132 for 6 h. Statistical analysis was performed via two‐tailed unpaired Student's *t*‐tests. Data are presented as mean ± SD; ns: not significant. All data is representative of three independent experiments.

Having established that APOL2 stabilizes Ku80 through proteasomal regulation, we next explored its effect on Ku80 ubiquitination. Ectopic expression of APOL2 was found to markedly decrease polyubiquitinated Ku80 levels, indicating a potential role for APOL2 in modulating Ku80's post‐translational modifications (**Figure** **4**A). Given that K48‐linked and K63‐linked ubiquitin chains represent two major forms of polyubiquitination involved in proteasomal degradation,^[^ ^23^ ^]^ we sought to delineate the specific ubiquitin linkage involved in Ku80 ubiquitination by expressing HA‐tagged ubiquitin variants (wild‐type, K48‐only, and K63‐only). Notably, APOL2 mainly diminished K48‐linked polyubiquitination while sparing K63‐linked chains (Figure 4B).

Our APOL2 interactome data showed that several well‐characterized deubiquitinating enzymes (DUBs),^[^ ^27^ ^]^ including USP7, USP10, USP16, USP40, and USP54, may interact with APOL2 (Figure S9A, Supporting Information). It has been reported that Ku80 strongly interacts with USP7.^[^ ^28^ ^]^ We hypothesize that APOL2 might suppress Ku80 ubiquitination via USP7. Consistent with the LC‐MS data, our Co‐IP data confirmed that USP7 interacts with APOL2 and Ku80 in both endogenous and exogenous contexts (Figure 4C,D; Figure S9B,C, Supporting Information). We next investigated whether USP7 affects the stability of Ku80. Western blot results demonstrated that Ku80 protein level increased concomitantly with USP7 elevation, while quantitative qRT‐PCR confirmed this regulation occurred independently of transcriptional changes (Figure 4E,F). Protein stability assays using CHX revealed that USP7 depletion could significantly reduce the half‐life of the Ku80 protein, whereas USP7 overexpression can prolong the half‐life of the Ku80 protein (Figure 4G). Consistent with its deubiquitinase function, USP7 overexpression reduced Ku80 ubiquitination, indicating USP7 stabilizes Ku80 through post‐translational deubiquitination (Figure 4H). Notably, knockdown of USP7 attenuated the ability of APOL2 to suppress Ku80 ubiquitination (Figure 4I), suggesting that USP7 serves as the essential effector mediating APOL2‐dependent regulation of Ku80 stability through deubiquitination.

### 2.6 APOL2 Promotes NHEJ Repair Through Ku80

**Figure 5:**
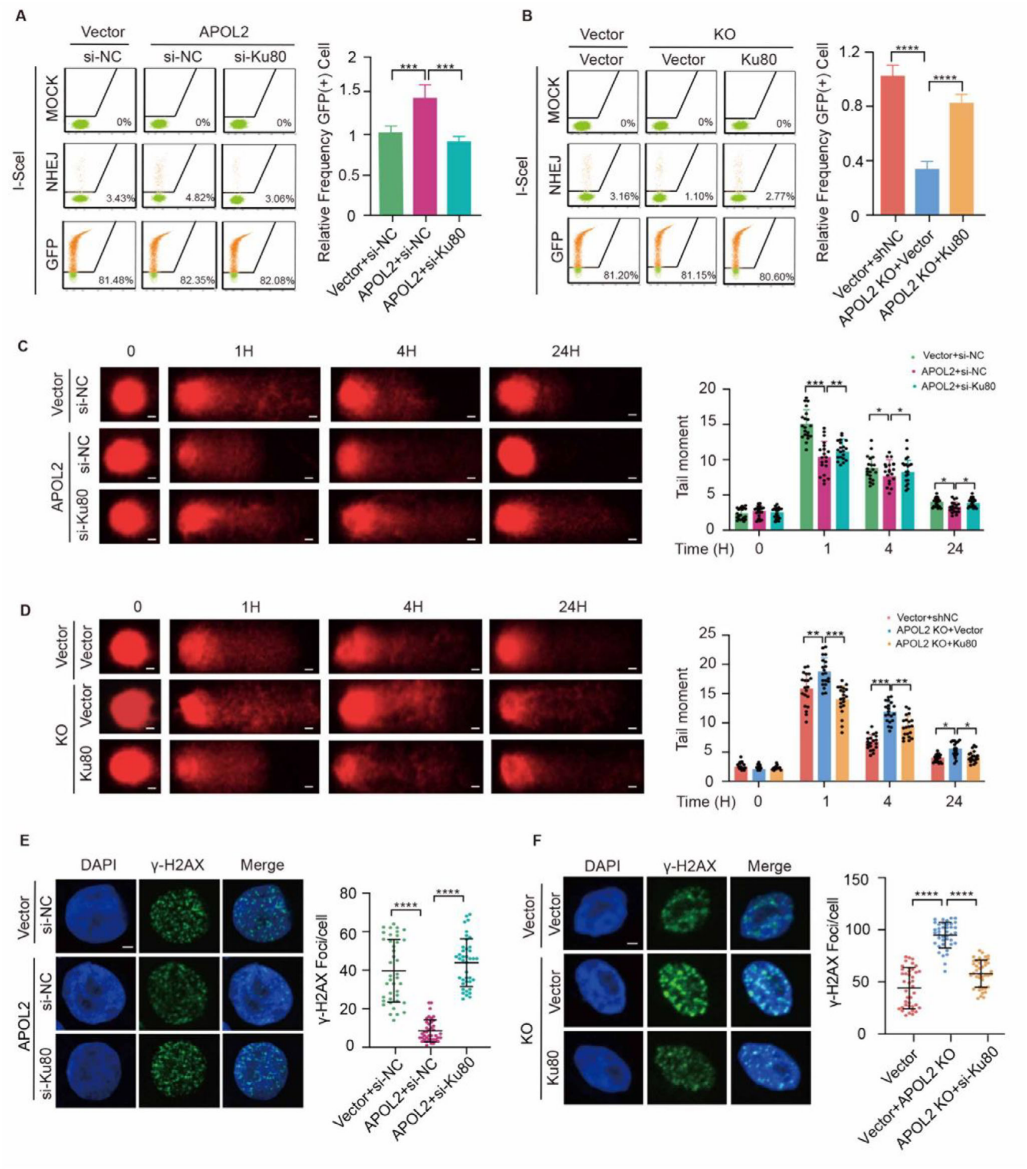
APOL2 promotes NHEJ repair through Ku80. A) NHEJ reporter assay in APOL2‐OE cells. Cells were transfected with the hEJ5‐GFP reporter plasmid and indicated constructs (Vector+si‐NC, APOL2+si‐NC, and APOL2+si‐Ku80), followed by infection with I‐SceI adenovirus to induce DNA DSBs. NHEJ repair efficiency was quantified via flow cytometry analysis of GFP‐positive cells 48 h post‐infection (*n* = 3). B) NHEJ reporter assay in APOL2‐KO cells. Experimental groups: shNC+vector, APOL2‐KO+vector, and APOL2‐KO+Ku80. Analysis was performed as in A). C) Comet assay in APOL2‐OE cells after 3 Gy IR. Representative images of comet assay and tail moment quantification of: i) Vector+si‐NC, ii) APOL2+si‐NC, and iii) APOL2+si‐Ku80 groups of MKN1 cells (*n* = 15). Scale bar, 10 µm. D) Comet assay in APOL2‐KO cells after IR. Experimental groups: i) shNC+vector, ii) APOL2‐KO+vector, and iii) APOL2‐KO+Ku80 groups of HGC27 cells. Scale bar, 10 µm. Analysis was performed as described in C). E) γ‐H2AX foci formation in APOL2‐OE cells after 3 Gy irradiation. Representative immunofluorescence images showing γ‐H2AX (green) and DAPI (blue) in shNC+vector, APOL2‐KO+vector, and APOL2‐KO+Ku80 groups of cells (left). Quantitative analysis of γ‐H2AX foci per cell (*n* = 40, right). Scale bar, 10 µm. F) γ‐H2AX foci formation in APOL2‐KO cells after 3 Gy irradiation. Experimental groups: i) Vector+si‐NC, ii) APOL2+si‐NC, iii) APOL2+si‐Ku80 groups. Scale bar, 10 µm. Analysis was performed as in E). All experiments were performed in triplicate. Error bars represent mean ± SD. Statistical analysis was performed via two‐tailed unpaired Student's t‐tests (A‐B, E‐F) or two‐way ANOVA (C‐D). Data was presented as mean ± SD; ^*^ *P* < 0.05, ^**^ *P* < 0.01, ^***^ *P* < 0.001, ^****^ *P* < 0.0001. All data are representative of three independent experiments.

To investigate whether APOL2‐mediated NHEJ repair requires Ku80, we performed EJ5‐GFP reporter assay to assess the NHEJ activity. We found that overexpression of APOL2 significantly increased the proportion of GFP‐positive cells, indicating enhanced NHEJ efficiency. Critically, this effect was abolished by Ku80 knockdown, thereby confirming Ku80's essential role in APOL2‐mediated NHEJ repair (**Figure** **5**A). In line with these findings, complementary loss‐of‐function experiments showed that APOL2 knockdown significantly reduced the proportion of GFP‐positive cells, indicating weakened NHEJ repair activity, which was substantially rescued by Ku80 re‐expression (Figure 5B). We next performed comet assays at 24 h after IR treatment and found that APOL2 overexpression significantly reduced tail moment, demonstrating accelerated DNA repair, while depletion of Ku80 reversed APOL2‐induced DNA repair (Figure 5C). Mirroring this, the elevated tail moment in APOL2 knockout cells, which is indicative of persistent DNA damage, was significantly reduced upon Ku80 reconstitution, indicating that DNA damage repair was impaired by APOL2 attenuation, while overexpression of Ku80 restored the repair ability (Figure 5D). Consistent with the comet assay results, immunofluorescence analysis of γ‐H2AX foci formation provided direct visualization of DSB repair kinetics. Radiation‐induced γ‐H2AX foci were significantly reduced in APOL2‐OE cells compared with control cells, while APOL2 knockout increased foci persistence. Importantly, Ku80 knockdown abolished the protective effect of APOL2 overexpression. Conversely, exogenous expression of Ku80 significantly rescued the repair defect in APOL2‐KO cells (Figure 5E,F). This bidirectional genetic evidence confirms that APOL2 depends on Ku80 to alleviate IR‐induced DNA damage.

Collectively, these reciprocal gain and loss of‐ function studies unequivocally demonstrate that APOL2 promotes NHEJ‐mediated DSB repair via Ku80.

### 2.7 APOL2 Confers Radioresistance In Vivo

To assess the effect of APOL2 on RT efficacy in vivo, we established a subcutaneous xenograft model. Local RT was administered after tumor formation (8 Gy fraction), followed by tumor size measurements every 4 days until the mice were euthanized at 28 days for further analysis (Figure S10A, Supporting Information). The overexpression efficiency of APOL2 protein in xenograft tumors was confirmed by Western blot (Figure S10B, Supporting Information). Compared with those in the control group, the tumors in the APOL2‐OE group exhibited greater growth patterns in terms of size, volume, and weight after IR treatment (Figure S10C–E, Supporting Information). Ki67 staining revealed that the proliferation rate of tumor cells in the APOL2‐OE group was significantly greater than that in the control group, while γH2AX expression level was lower in the APOL2‐OE group than that in the control group upon IR treatment (Figure S10F,G, Supporting Information). Furthermore, we examined key components involved in the NHEJ pathway. IHC analysis revealed significantly elevated Ku80 and p‐DNA‐PKcs protein levels in APOL2‐OE tumors compared with the control group, suggesting high NHEJ repair activity upon IR treatment in APOL2‐OE tumors (Figure S10H,I, Supporting Information). These data suggest that APOL2 overexpression enhances radioresistance in GC.

**Figure 6:**
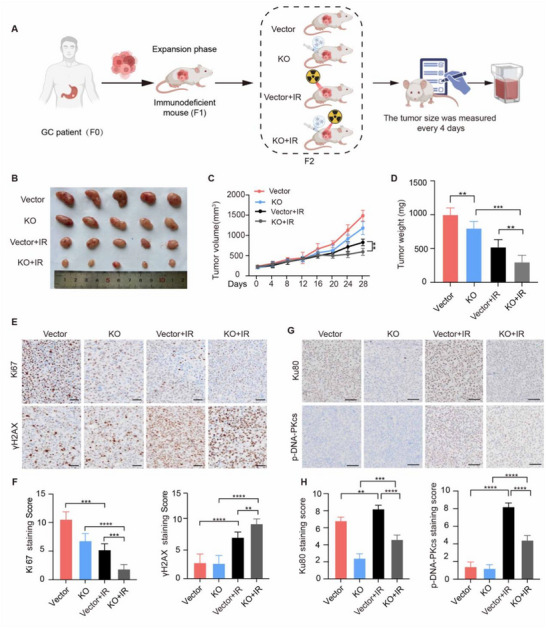
APOL2 confers radioresistance in vivo. A) Schematic of the PDX model construction and treatment protocol. B) Representative images of different groups of excised xenograft tumors 28 days after 8 Gy local RT at the endpoint. C‐D) Xenograft tumor growth curves (C) and endpoint weight (D) for each treatment group (*n* = 5). E–H) Representative IHC images and quantitative analysis of Ki67, γ‐H2AX, Ku80 and p‐DNA‐PKcs in tumor tissue sections (*n* = 5). E‐F: Scale bar, 50 µm. G‐H: Scale bar, 100 µm. Statistical analysis was performed via two‐tailed unpaired Student's t‐tests (D, F‐H) or two‐way ANOVA (C). Data was presented as mean ± SD; ^**^ *P* < 0.01, ^***^ *P* < 0.001, ^****^ *P* < 0.0001. All data are representative of three independent experiments.

To further confirm the role of APOL2 in radioresistance, we established a subcutaneous patient‐derived xenograft (PDX) model in mice. After the PDX model was established, lentiviruses carrying APOL2 knockout vectors were injected into subcutaneous tumors. 48 h after injection, local RT was administered to the tumor area (8 Gy per fraction per week, total 1 fraction) (**Figure** **6**A). The APOL2 knockout (APOL2 KO) efficiency in the tumors was confirmed by western blot (Figure S10B, Supporting Information). Compared with those in the control group, the tumors in the APOL2 KO group exhibited a weaker growth pattern in terms of size, volume, and weight after IR treatment. Tumor growth was fastest in the vector group without any intervention, while tumor growth was significantly inhibited in the APOL2‐KO plus IR group (Figure 6B–D). IHC analysis revealed lower Ki67 expression and higher γH2AX expression in the KO group than that in the control group upon the IR treatment (Figure 6E,F). We also measured the expression of Ku80 and p‐DNA‐PKcs in tumors. In line with previous results, the expression of Ku80 and p‐DNA‐PKcs was significantly lower in APOL2‐KO tumors compared with the control group tumors (Figure 6G,H). These results demonstrate that APOL2 potentiates NHEJ activity to confer radioresistance in vivo, making it a promising therapeutic target for radiosensitization in GC.

### 2.8 Formononetin Restores Radiosensitivity by Suppressing APOL2‐Ku80 Interactions

**Figure 7:**
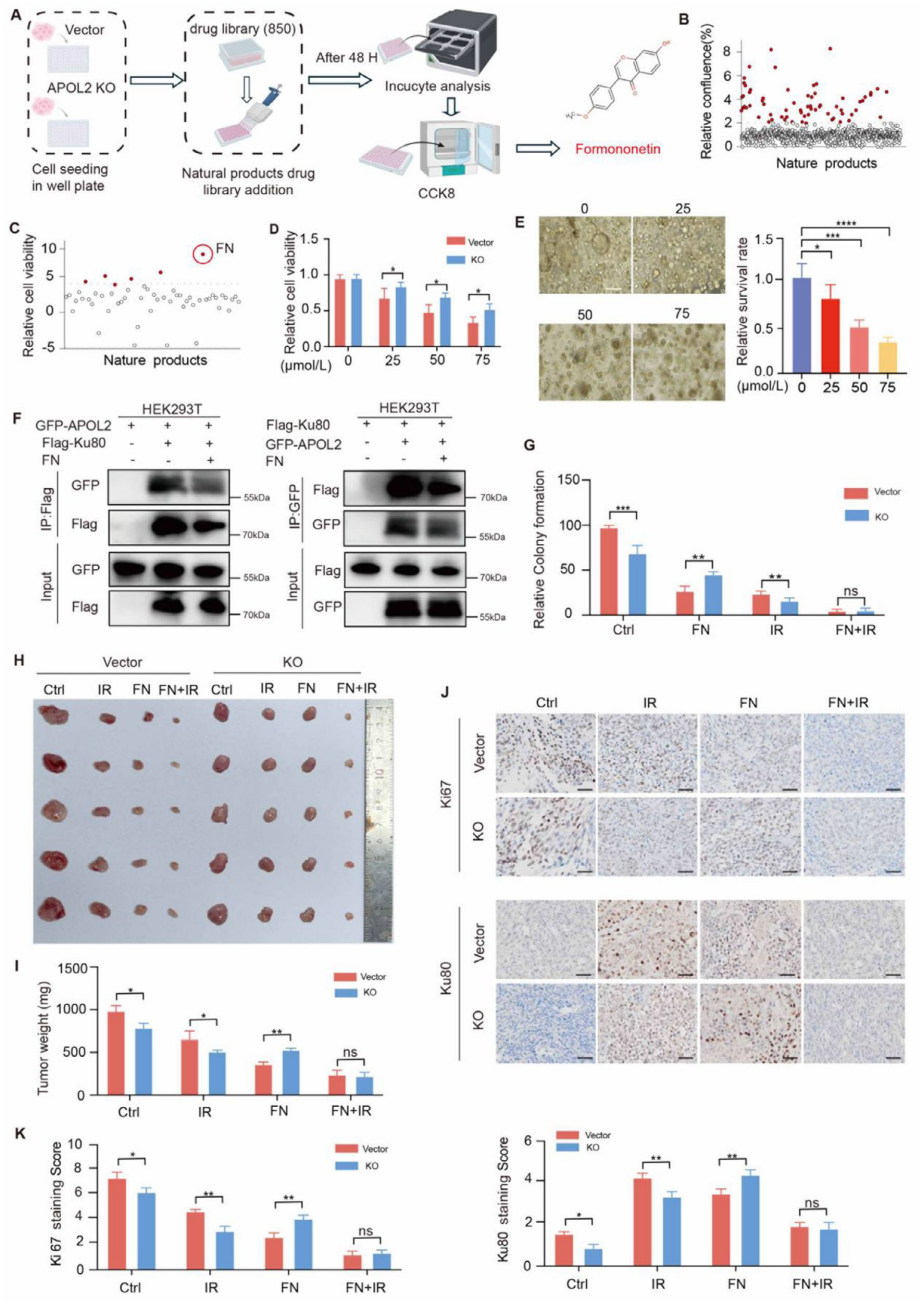
FN restores radiosensitivity by disrupting APOL2‐Ku80 interactions. A) Schematic diagram of the high‐throughput screening workflow of for 850 natural compounds in WT and APOL2‐KO cells. B) Scatter plot showing the 65 natural products initially screened and identified. Relative convergence was calculated as (normalized survival rate of KO group)/(normalized survival rate of the WT group) × 100%, where the survival rate values were first normalized to their respective untreated control groups (set to 100%). Each dot represents a drug, and the red dots respectively indicate the threshold data of sensitive drugs in APOL2‐KO cells. C) The relative cell viability of vector and APOL2‐KO cells treated with 65 candidate natural products for 48 h was quantified by CCK‐8 method. D) Bar graph showing the relative viability (%) of WT (vector) and APOL2‐KO cells treated with FN at the indicated concentrations (0, 25, 50, 75 µm) for 48 h, as determined by CCK‐8 method (n = 3). E) FN‐mediated disruption of patient‐derived GC organoids. Representative bright‐field images showing organoid structural integrity after 48 h treatment with different concentrations of FN (0‐50 µm, left). Scale, 10 µm. Quantitative analysis of the organoid relative survival rate was measured by ImageJ. Data normalized to the control group (set as 100%). F) Co‐IP was used to detect the interaction between APOL2 and Ku80 in HEK293T cells treated with 50 µm FN for 48 h. G) Statistical graphs of the colony formation in vector and APOL2‐KO cells after IR and FN treatment (*n* = 3). H) Representative images of excised xenograft tumors at the endpoint (day 28 post‐treatment). Nude mice bearing subcutaneous tumors derived from WT or APOL2 KO GC cells were treated with: i) vehicle, ii) FN (20 mg/kg), (iii) RT (8 Gy), or (iv) combination therapy (*n* = 5). I) Quantitative analysis of tumor weights. Excised tumors were weighed after treatments as described in H). J,K) Representative IHC staining images (J) and quantitative analysis (K) of Ki67, γ‐H2AX, and Ku80. Scale bar, 100 µm. Statistical analysis was performed via two‐tailed unpaired Student's *t*‐tests (E) or two‐way ANOVA (D, I, K). Data was presented as mean ± SD; ^*^ *P* < 0.05, ^**^ *P* < 0.01, ^***^ *P* < 0.001, ^****^ *P* < 0.0001. All data are representative of three independent experiments.

To address the challenge of APOL2‐mediated radioresistance in GC cells, we performed high‐throughput screening of a library of natural products containing more than 800 compounds to identify natural products that could selectively target APOL2 positive GC cells (**Figure** **7**A). IncuCyte analysis screening identified 65 potential candidates that exhibit selective cytotoxicity toward APOL2‐positive cells compared with APOL2‐KO cells (Figure 7B). CCK8 analysis further narrowed down these candidates and confirmed that FN exhibited the highest selective cytotoxicity toward APOL2‐positive cells among all the tested natural products (Figure 7C). FN decreased the viability of APOL2‐positive cells in a dose‐dependent manner, while APOL2‐KO cells exhibited less sensitivity to FN treatment (Figure 7D). GC organoid experiments also confirm the inhibitory effect of FN on GC (Figure 7E).

To explore whether FN affects the interaction between APOL2 and Ku80, Flag‐Ku80 and GFP‐APOL2 were transfected into HEK293T cells, and Co‐IP was performed after 48 h of FN treatment. We found that the interaction between APOL2 and Ku80 was attenuated following FN treatment (Figure 7F). Moreover, FN treatment effectively abrogated APOL2‐mediated radioresistance, resensitizing cells to IR (Figure 7G; Figure S11A, Supporting Information). Having established the therapeutic efficacy of FN in GC cells in vitro, we next investigated whether FN exerts its antitumor effects by targeting APOL2 in vivo. We established a subcutaneous tumor xenograft model by using WT cells and APOL2‐KO cells. After two weeks, when palpable tumor masses were formed, the mice were treated with or without IR, FN, or IR plus FN. Consistent with the in vitro data, the mouse experimental results revealed that FN significantly suppressed tumor formation in the control group. Moreover, FN could abolish APOL2‐mediated radioresistance in vivo (Figure 7H,I; Figure S11B, Supporting Information). Notably, upon FN treatment, compared with tumors from the APOL2‐KO group, the tumors from the control group showed higher expression of γH2AX and lower expression of Ki67 and Ku80. Moreover, FN could abolish APOL2‐induced Ku80 expression upon IR, leading to a high DNA damage level (Figure 7J,K; Figure S11C,D, Supporting Information). To assess the potential systemic toxicity of FN treatment, we performed histopathological analysis of major organs from tumor‐implanted mice via H&E staining. The results showed no significant morphological alterations (Figure S11E, Supporting Information). These findings demonstrate FN's potent and selective anti‐tumor efficacy of FN in vivo, with no observable toxicity to normal organs, highlighting its clinical potential for overcoming radioresistance in cancer therapy.

Collectively, our findings demonstrate that FN disrupts the APOL2‐Ku80 interaction, leading to Ku80 destabilization and decreased its expression, thereby abolishing APOL2‐mediated radioresistance and restoring tumor cell sensitivity to IR.

## 3 Discussion

RT remains a mainstay treatment for locally advanced and metastatic GC, particularly in combination with chemotherapy or as palliative care. However, intrinsic and acquired radioresistance, which is prevalent in GC, significantly limits its clinical efficacy, contributing to cancer recurrence and poor prognosis.^[^ ^29^ ^]^ Therefore, addressing the radiosensitivity of GC cells is a critical issue. Amongst the several abnormal genes that have been reported as potential prognostic biomarkers for GC as they play important roles in its development and progression,^[^ ^30^ ^]^ APOL2 has been implicated in cancer invasion and metastasis.^[^ ^21^ ^]^ In addition, some studies have revealed that APOL2 regulates tumor cell proliferation, apoptosis, and drug resistance.^[^ ^18^ ^]^ Nonetheless, research specifically focusing on APOL2 in GC is lacking.

Elucidating the mechanisms by which APOL2 drives GC progression may provide valuable insights for the development of new therapeutic strategies. In our study, clinical analyses revealed the prognostic significance of APOL2, with TCGA data and our institutional samples consistently showing high APOL2 expression in GC tissues, and high APOL2 overexpression correlating with advanced clinical stages and poor prognosis. IHC and Western blot assays confirmed these findings, revealing elevated APOL2 levels in tumor tissues.

This clinical association prompted mechanistic investigations into APOL2's functional contributions. Proteomic and KEGG pathway analyses revealed a strong link between APOL2 and the DNA damage repair pathway. APOL2 specifically enhances NHEJ efficiency, as demonstrated by its ability to stabilize Ku80 by suppressing its ubiquitination, a critical regulator in NHEJ repair, to initiate DSB repair. Ubiquitination serves as a crucial post‐translational modification that regulates protein stability, localization, and activity.^[^ ^31^ ^]^ This modification process is highly dynamic and reversible, with DUBs acting to hydrolyze ubiquitin chains or monomers and counterbalance ubiquitination signals.^[^ ^32^ ^]^ Among the diverse DUB families, ubiquitin‐specific proteases (USPs) represent the largest and most structurally diverse group, comprising ≈60 members in humans.^[^ ^33^ ^]^ USP7 is an important member of the USP family. It plays a significant role in tumorigenesis, development, metastasis, and treatment resistance by regulating the ubiquitination levels of multiple key proteins.^[^ ^34^ ^]^ Our r findings indicate that USP7 interacts with APOL2 and Ku80, and is involved in APOL2‐mediated Ku80 stability. Moreover, APOL2 strictly requires Ku80 to enhance NHEJ repair, thereby conferring radioresistance.

Although we identified Ku80 as an important medium for APOL2‐driven NHEJ repair in vitro, the role of this axis in vivo remains unknown. We subsequently constructed PDX and CDX models to further explore the therapeutic effect of APOL2 in vivo. At present, the clinical radiotherapy for gastric cancer mainly adopts a graded regimen. However, in specific cases related to gastric cancer, doses exceeding 5 Gy are increasingly used, and a single high dose can be directly administered to the tumor bed.^[^ ^35^ ^]^ In the animal models used in this study, we found that the significant differences observed between the 0 Gy and 8 Gy groups at key endpoints (delayed tumor growth, survival benefit, apoptotic index, and biomarker changes) clearly indicate that there is a substantial biological dose‐response effect at the 8 Gy dose.

During the evolution of cancer cells, multiple integrated molecular signals lead to increased resistance of tumor cells to RT, resulting in RT failure. Certain chemicals, such as cisplatin and paclitaxel, are used as radiosensitizers and can increase the sensitivity of tumor cells to RT.^[^ ^36^ ^]^ In this study, we identified FN as a potential therapeutic agent by high‐throughput drug screening of an approved natural product drug library. FN, a phytoestrogen derived from red clover, plays important roles in disease management by regulating inflammation, angiogenesis, the cell cycle, and apoptosis.^[^ ^37^ ^]^ It has been reported that FN can regulate different signal transduction pathways in cancer, such as transcriptional signal transduction and activator factor 3 (STAT3), phosphatidylinositol 3 kinase/protein kinase B (PI3K/Akt), and mitogen‐activated protein kinase (MAPK), to exert its antitumor effects.^[^ ^38^ ^]^ However, the therapeutic potential of FN in GC has not been reported. In this study, we found FN could overcome APOL2‐mediated radioresistance and suppress the interaction between APOL2 and Ku80. We believe that FN may inhibit the APOL2‐induced NHEJ repair activity, ultimately restoring radiosensitivity. The precise molecular mechanism of this interaction remains to be elucidated. Future studies will focus on elucidating the intricate mechanisms of FN action. In addition, we plan to optimize the molecular structure of FN through future chemical modification to enhance its efficacy and reduce the required effective concentration.

**Figure 8:**
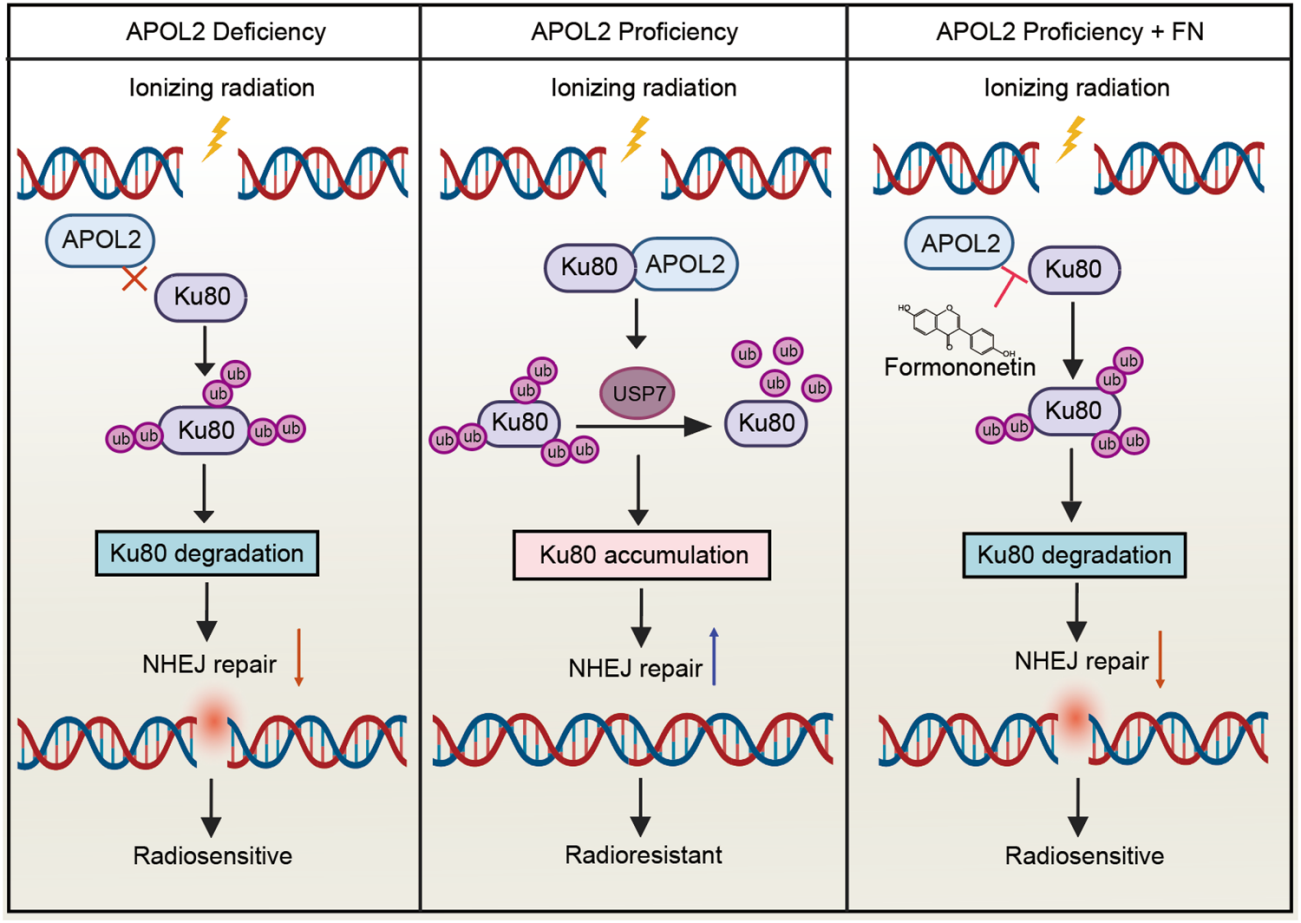
Schematic diagram of the mechanism by which APOL2 enhances radioresistance in GC. APOL2 stabilizes Ku80 through USP7‐mediated deubiquitination, thereby facilitating NHEJ‐mediated DNA repair and enhancing the radioresistance of GC. FN can suppress the interaction between APOL2 and Ku80, reducing radioresistance.

Taken together, these findings suggest that elevated APOL2 expression is associated with poor prognosis in GC patients. Furthermore, APOL2 has been shown to reduce IR‐induced DNA damage and enhance the radioresistance of GC cells. Mechanistically, APOL2 stabilizes Ku80 through USP7‐dependent deubiquitination, resulting in Ku80 accumulation, which enhances NHEJ repair efficiency and subsequently confers cellular radioresistance (Figure 8). Our study provides new insights into the mechanism underlying radioresistance in GC cells and proposes a new solution to address the dilemma of radioresistance in GC (Figure 8).

## 4 Experimental Section

### Cell Culture

Human cell lines AGS (Cat No. CRL‐1739), SNU1 (Cat No. CRL‐5971), NCI‐N87 (Cat No. CRL‐5822), and HEK293T (Cat No. CRL‐3216) were obtained from the American Type Culture Collection (ATCC). The human cell line HGC27 (Cat No. TCHu22) was obtained from the National Collection of Authenticated Cell Cultures. The human cell lines MKN1 (Cat No. FH0320), NUGC3 (Cat No. FH1016), NUGC4 (Cat No. FH0480), MKN45 (Cat No. FH‐MKN‐45), and GES‐1 (Cat No. FH0273) were obtained from Shanghai Fuheng Biological Co., Ltd. The MKN1, NUGC3, NUGC4, GES‐1, NCI‐N87, AGS, and HGC27 GC cell lines were cultured in RPMI‐1640 (HyClone, #SH30022.01B) supplemented with 10% fetal bovine serum (ExCell Biol, #FSP500). MKN45, SUN‐1, and HEK293T cells were maintained in DMEM (HyClone, SH30243.01B) supplemented with the addition of 10% fetal bovine serum. All media were further supplemented with 1% penicillin‐streptomycin (Cytiva, #SV30010) to prevent contamination. Cells were incubated in a humidified incubator containing 5% CO_2_ at 37 °C.

### Ionizing Radiation

Cell irradiation doses and times were 1 Gy (58s), 3 Gy (176s), 5 Gy (294s), and 7 Gy (411s), with a dose rate of 17 mA (Rad Source, RS2000 SUPER). For animal irradiation, we used the SARRP small animal irradiator with the following parameters: gantry angle 90°, collimator angle 0°, SSD technique with 350 mm source‐to‐skin distance, 10 × 10 mm field size, 1.5 mm copper filter, 8 Gy dose, 220 kV voltage, 13 mA current, and dose rate of 4 Gy/min.

### Plasmid Construction

The pCMV‐GFP‐APOL2 (WT) and pCMV‐GFP‐APOL2 (OE) plasmids were purchased from Genechem Biotechnology Co., Ltd. (Shanghai, China). pDR‐GFP (#26475), hprt‐SA‐GFP (#41594), pim‐EJ5‐GFP (#44026), EJ2‐GFP‐puro (#44025), and I‐SceIg were purchased from Addgene Co., Ltd. (USA). The pCMV‐GFP‐APOL2 (aa 1–68) (G43051), pCMV‐GFP‐APOL2 (aa 1–111) (G43053), pCMV‐GFP‐APOL2 (aa 1–140) (G43052), pCMV‐GFP‐APOL2 (aa 130–260) (G43054), pCMV‐GFP‐APOL2 (aa 240–337) (G43056) and pCMV‐Myc‐USP7 (P48323) plasmids were obtained from Miaoling Biotechnology Co., Ltd. (Wuhan, China). The plasmids pCMV‐His‐Ub (K48 only) and pCMV‐His‐Ub (K63 only) were provided by Prof. Jing Ji from the Hangzhou Institute of Medicine, Chinese Academy of Sciences (Hangzhou, China). Small interfering RNAs (siRNAs) targeting APOL2 and negative control siRNA (si‐NC) were obtained from Genomeditech Biotechnology Co., Ltd. (Shanghai, China). siRNAs targeting si‐USP7/si‐Ku80 and si‐NC were obtained from Miaoling Biotechnology Co., Ltd. (Wuhan, China). The siRNA sequences are detailed in Table S1 (Supporting Information).

### Plasmid Transfection and Viral Transduction

HEK293T cells were transfected with plasmid DNA using a Jetprime transfection kit (Polyplus, #101000046) following the manufacturer's instructions. Lentiviral supernatant was collected 48 h post‐transfection and subsequently used to infect GC cells. Infected cells were selected with puromycin (1 µg mL^−1^) (Beyotime, #ST551) 48 h after infection. The transfection efficiency of the target gene was detected by Western blot.

### qRT‒PCR

Total RNA was extracted using a Fastagen kit (Yishan, #RN001), and reverse transcribed into cDNA using the reverse transcription kit (TaKaR, #RR036A). Quantitative PCR was performed with the SYBR Premix Ex TaqTM II kit (TaKaRa, #RR086A). Relative gene expression levels were normalized to GAPDH as a control and calculated using the 2^−ΔΔCT^ method. The sequence of primers used for the RT‒qPCR assays were listed in Table S1 (Supporting Information).

### Lentivirus Packaging and Stable Cell Line Construction

For lentivirus packaging, HEK293T cells in the logarithmic growth phase were seeded (1 × 10^7^) in a 10 cm petri dish (BIOFIL, # TCD010100). The plasmids encoding GFP and GFP‐APOL2‐OE were transfected into MKN1 cells, and 1 µg mL^−1^ puromycin was added for selection after 24 h. Fluorescent cells were observed under fluorescence microscopy. The cells that survived for 96 h were regarded as stable strains. The APOL2‐KO plasmid was transfected into HEK293T cells, and the culture supernatant of APOL2‐KO cells was collected 48 and 72 h after transfection. The collected virus supernatant was centrifuged at 3500 rpm for 10 min and filtered with a 0.22 µm filter (Merck Millipore, SLGP033RB). The virus was added to HGC27 cells in the logarithmic growth phase when the cells reached a confluence of 60–70%. A total of 5 µg/mL polystyrene (Macklin, # 9003‐53‐6) was added to the medium for 48 and 72 h, and 1 µg mL^−1^ puromycin was added for selection. The strains that survived for 96 h were considered stable strains.

### CCK8 Assay

MKN1 or HGC27 cells were seeded into 96‐well plates (NEST,#701001) at a density of 3000 cells/well, with each well containing 100 µL culture medium. After overnight incubation to allow cell attachment, the cells were treated with 1 Gy, 3 Gy, 5 Gy, or 7 Gy IR. After 24 and 72 h the supernatant was discarded, and CCK‐8 solution (DOJINDO, CK04) was added to each well to assess cell viability. The optical density (OD) of each well was measured at 450 nm. Relative cell viability (%) was calculated as follows: viability (%) = (OD_experiment_−OD_blank_)/(OD_control_−OD_blank_) ×100%.

### Mass Spectrometry

To identify APOL2‐interacting proteins by mass spectrometry, we collected cells from the NC and APOL2‐overexpression groups. Then, GFP magnetic beads were subsequently immunoprecipitation, SDS‐PAGE separation, and Coomassie blue colloid staining. The magnetic beads used for protein incubation were sent to the central laboratory of Zhejiang Cancer Hospital, and high‐performance liquid chromatography was performed to detect the APOL2 interactome.

### Western Blot

Total protein was extracted with RIPA lysis buffer (Beyotime, #P1003K), and protein concentration was quantified using a BCA protein assay kit (Beyotime, #3P0011). Proteins were separated on 10% or 12% SDS‒PAGE gels and then transferred onto PVDF membranes (Millipore, #BM4AB8360A). The membrane was incubated with APOL2 (Proteintech, #25925‐1‐AP), Ku80 (Proteintech, #16389‐1‐AP), ubiquitin antibodies (Proteintech, #10201‐2‐AP), Ku70 (Affinity, #AF0300), USP7 (Abcam, #ab108931), and β‐actin (Proteintech, #20536‐1‐AP) antibodies at 4°C overnight. The membranes were washed three times with TBS (Servicebio,G0001‐2L)/0.1% Tween 20 (ZHANYUN, 9005‐64‐5) (10 min per wash), followed by incubation with HRP antibody (Proteintech, SA0000‐1) for 2 h. Protein bands were visualized via a chemiluminescence detection system.

### Colony Formation Assay

MKN1 and HGC27 cells were seeded in a 6‐well plate (NEST, #703001) at a density of 800/well. The cells were treated with 1 Gy, 3 Gy, 5 Gy, or 7 Gy IR after adhesion. After 2 weeks, cells were fixed with 4% paraformaldehyde (Beyotime, #P0099) and stained with crystal violet (Beyotime, #C0121). The plating efficiency (PE) was calculated as follows:PE (%) = (number of clones in the control group/number of cells seeded in the control group) ×100.

### Immunofluorescence Staining

MKN1 and HGC27 cells were seeded on cell culture coverslips in 24‐well plates (NEST, #702001). Once the cells reached a confluence of ≈70% confluence, they were subjected to 1 Gy, 3 Gy, 5 Gy or 7 Gy IR treatment. Cells were then fixed with 4% paraformaldehyde (Beyotime, #P0099) for 20 min and permeabilized with 0.5% PBST (0.15 mL Triton X‐100 + 50 mL PBS; Macklin, # 9002‐93‐1) for 30 min. Then, the cells were blocked with an immunofluorescence blocking solution (BIOESN, #BES21694KB) for 1 h and incubated overnight at 4 °C with antibodies against γH2AX (CST, #7631T), Ku80 (Affinity, #BF0183), Ku70, DNA‐PKcs (Affinity, #AF5340) and p‐DNA‐PKcs(Affinity, #AF3360) diluted at a ratio of 1:200. The next day, cells were washed three times with PBS (5 min per wash) and then incubated with a fluorescently labeled secondary antibody (Abcam, #150077) for 1 h at room temperature in the dark. Finally, the cells were mounted with DAPI (Beyotime, #C1005) and imaged under a confocal laser microscope.

### Chromosomal Aberration

The GC cell lines MKN1 and HGC27 were seeded in 10 cm well plates and treated with 3 Gy IR upon reaching a confluence of 60%. After 24 h, colchicine (0.1 µg mL^−1^; Nebula Bioscience, CAS#64‐86‐8) was added, and the cells were treated for 4 h. After harvesting, the cell suspension was centrifuged, and ≈1 mL of supernatant was retained to resuspend the cell pellet. A KCl hypotonic solution (0.075 M; Macklin, #7447‐40‐7) was added to top up to a final volume to 5–6 mL. The mixture was incubated in a 37 °C water bath for 30 min to induce cell swelling. The process was terminated by adding 1 mL of a 3:1 methanol:acetic acid fixative. After incubation for 5 min, the cells were centrifuged at 900 rpm, washed three times with fresh fixing solution, and then resuspended with drops added on the slide. The cells were incubated with Giemsa stain for 20 min. The samples were dried on a 65 °C heat block, observed under a light microscope, and photographed. All aberrations were scored according to published classification standards.^[^ ^39^ ^]^

### Chromosomal Lagging

The GC cell lines MKN1 and HGC27 were seeded in a 24‐well plate and treated with 3 Gy IR upon reaching 50% confluence. After the culture medium was discarded, the cells were washed twice with precooled PBS, followed by fixation with 4% paraformaldehyde for 20 min. Finally, the cells were mounted with DAPI, observed, and photographed under a laser confocal microscope after 5 min incubation. The frequency of lagging chromosomes (the percentage of lagging chromosomes in late‐stage cells) was quantified using the previously reported method.^[^ ^40^ ^]^

### Micronucleus Assays

MKN1 and HGC27 cells were seeded on the cover plate of a 24‐well plate and treated with 3 Gy IR when the convergence degree reached 50%. After 24 h, 1 µg mL^−1^ cytochalasin B (MCE, #HY‐16928) was added for treatment. After 48 h, the culture medium was discarded and the cells were washed twice with pre‐cooled PBS, before fixing with 4% paraformaldehyde for 20 min. Then, the cells were washed thrice with pre‐cooled PBS for 5 min each time. Subsequently, the cells were embedded with DAPI, incubated for 5 min, and then observed and photographed under a laser confocal microscope. Micronuclei were identified manually by distinct DAPI staining outside the main nucleus.^[^ ^41^ ^]^

### Comet Assay

A comet electrophoresis kit (Beyotime, #C2041M) was used to evaluate 3 Gy IR‐induced cellular DNA damage. After 8 Gy X‐ray irradiation, the cells were collected and adjusted to a density of 1 × 10^6^ cells/mL. The cells were then mixed with comet agarose and transferred onto comet slides. The slides were then incubated in precooled neutral lysis buffer containing DMSO (Meilunbio, # PWL064) at 4 °C for 2 h. Electrophoresis was performed at 4 °C at 25 V for 30 min under neutral conditions. The samples were then stained in the dark with Vista Green DNA Dye for 15 min and subsequently examined under a Leica inverted fluorescence microscope. The tail moment was quantified using CASP version 1.2.3 beta2 (CaspLab, Wroclaw, Poland). and 15 or 20 cells were scored for each case.

### In Vitro Pull‐Down Assay

Plasmids encoding GST and GST‐APOL2 were transformed into BL21 competent cells and induced with isopropyl β‐D‐thiogalactoside (IPTG) (1 mm) at 18 °C for 16 h. The cells were lysed in lysis buffer (20 mm Tris‐HCl, 200 mm NaCl, 5% glycerol, and 0.3% Triton X‐100) and the proteins were purified through affinity chromatography using a matrix (Transgen Biotech, DP201‐01) followed by glutathione (10 mM). HEK293T cells were transfected with plasmids encoding Flag‐Ku80 or Flag‐Ku70. Cells were lysed with NP‐40 lysis buffer, and the cell lysates were immunoprecipitated with anti‐Flag agarose (Sigma, A4596). Flag‐Ku80 or Flag‐Ku70 was eluted with 3xFLAG peptide (100 mg mL^−1^ in PBS) (Sigma, SAE0194). Purified GST or GST‐APOL2 (5 µg) was incubated overnight with Flag‐Ku80 or Flag‐Ku70 at 4 °C, and then glutathione agarose (Transgen Biotech, DP501‐01) was pulled down in PBS containing protease inhibitors for 2 h. Glutathione agarose was washed three times with PBS, and a Western blot was performed.

### Immunoprecipitation (Co‐IP) Assay and Mass Spectrometry

Cells were lysed in IP buffer (APPLYGEN, #C1504) and then hybridized with anti‐GFP magnetic beads (Beyotime, P2132). After washing with cold PBS, the proteins were pelleted through centrifugation and then resuspended in 1x SDS‒PAGE loading buffer. The proteins were denatured by boiling for 5 min before SDS‒PAGE, followed by transfer to a PVDF membrane. PVDF membranes were blocked with BSA for 2 h, followed by incubation with the primary antibody overnight at 4 °C. The membranes were washed three times with 1×TBS/0.1% Tween‐20 (10 min per wash), followed by incubation with the secondary antibody at room temperature for 1 h. The proteins of interest were visualized using enhanced chemiluminescence (ECL) reagents (Beyotime, #P0018S). For mass spectrometry analysis, MKN1 cells were transfected with GFP‐APOL2 or GFP‐Vector plasmids to construct stable cell lines. Whole‐cell lysates were prepared and subjected to Co‐IP. The immunoprecipitated samples were then analyzed via mass spectrometry in the central laboratory of Zhejiang Cancer Hospital.

### HR, NHEJ, MMEJ and Single Strand Annealing (SSA) Reporter Assays

3 × 10^5^ HEK293 T cells were first seeded in a 6‐well plate. The following day, cells were transfected with hDR‐GFP, pim‐EJ5‐GFP plasmids, EJ2‐GFP‐puro plasmids or SA‐GFP using jetPRIME, followed by medium change after 6 h. On the third day, cells were selected with puromycin (2 µg mL^−1^) for 48 h, then reseeded in 6‐well plates at a density of 3 × 10^5^ cells/well. After 24 h, cells were transfected with either I‐SceI plasmid, siAPOL2‐1 plasmid, siAPOL2‐2 plasmid, siAPOL2‐3 or si‐Ku80 plasmid. Finally, 48 h later, cells were collected, and the percentage of GFP‐positive cells was obtained determined via flow cytometry.

### Ubiquitination Analysis

Cells transfected with HA‐Ub, GFP‐APOL2, Flag‐Ku80, Myc‐USP7, or other indicator plasmids were treated with 10 µm MG132 (MCE, # HY‐13259) for 6 h. Following treatment, the cell lysates were subjected to immunoprecipitation according to the Co‐IP method described above.

### Nude Mouse Tumor Model

Four‐week‐old BALB/c nude mice were divided into four groups: GFP, GFP+IR, GFP‐APOL2, and GFP‐APOL2+IR groups. A 0.2 mL cell suspension was injected subcutaneously into the left forelimb of the animals in the corresponding groups. After injection, the activity, eating, and mental state of the mice were monitored daily. After 14 days, the tumor‐bearing mice received local 8 Gy exposure in the pelvic cavity. The long (L) and short (S) of the transplanted tumors were measured with a Vernier caliper every 4 days. The mice were sacrificed 28 days later. The weights and sizes of the tumors were measured and photographed. Tumor formation was defined as the time at which the tumor diameter reached 0.5 cm. Tumor volume was calculated using the formula V (cm^3^) = (L × S^2^) × 0.5.

### PDX Model

Patient surgical samples were immersed in HBSS sample buffer and transferred to the laboratory. After the tumor tissue was removed, 1–2 immunodeficient NCG mice were inoculated according to the tumor volume and labeled the P0 generation. After inoculation, the daily behavior and clinical status of the animals were monitored regularly. Once tumor formation occurred, the L and S diameters of the tumors were measured regularly with Vernier calipers, and the weights of the mice were recorded with an electronic balance. The P0 generation tumors are expected to grow in animals for 2‒3 months. If the volume of P0 generation tumors grew to 100‒500 mm^3^, they were passaged to subsequent animals, which were designated the P1, P2, and P3 generations, respectively. The mice in the three‐generation patient‐derived xenograft (PDX) model were divided into four groups: NC, APOL2‐KO, NC+IR and Apol2‐KO+IR. Lentiviruses for intratumoral injection were collected using HEK293T cells and centrifuged at 30000 rpm for 4 h at 4 °C. Empty package viruses and APOL2‐KO viruses were injected at a dose of 1 × 10^8^ pfu/100 µL per mouse twice a week for 3 consecutive weeks. Tumor‐bearing mice received local exposure to ^Co^60 at a dose of 8 Gy. Tumor volume was measured every 4 days, and the mice were euthanized after 28 days. Tumor xenografts were excised, fixed, weighed, photographed, and stored for subsequent analyses. Tumor volume was calculated using the formula V (cm^3^) = (L × S^2^) × 0.5.

### Model Construction, Grouping, and Drug Administration

The expanded vector and APOL2‐KO cells were collected and inoculated subcutaneously into nude mice at a ratio of 8 × 10^6^ cells, with 5 nude mice in each group, for a total of 8 groups. Two weeks later, tumor formation in the mice was observed. Among them, one group was selected from the Ctrl group and another group from the APOL2‐KO group for treatment with FN alone (50 mg/kg) once a day. One group received RT alone at a dose of 8 Gy; A group of RT treatments combined with the FN treatment. FN were administered by gavage, while the control group was given the same amount of normal saline in the same way. Tumor volume was measured using a vernier caliper at a frequency of once every four days. After 28 days, nude mice were euthanized, the subcutaneous tumor masses and important organs were removed, and weighed. After collection, the tumor tissues and important organs were immersed in formalin fixative, and subsequent H&E and immunohistochemical staining were performed.

### Organoid Culture

Organoid cultures were derived from GC biopsy samples. The tumor tissue was rinsed, chopped, and treated with collagenase digestion solution (0.1 mg mL^−1^; Roche, 11088858001) at 37 °C for 1 h. The digestion process was terminated with cold medium, and the mixture was filtered through a JET BIOFIL (CSS013100) with a 70 µm filter to obtain a cell suspension, which was then centrifuged at 400 g for 5 min. The collected cell particles were resuspended in 50% matrix (BD Biosciences, California, USA)/organoid medium and precoated with 50 µL drops of a 60% matrix in the middle of each well of a six‐well plate. The droplets were incubated at 37 °C and 5% CO_2_ for 30 min to cure. Once the droplets had solidified, 0.5 mL of organoid medium was added to each well, and the medium was replaced every 3–4 days. The survival rates of the organoids were assessed using the CCK‐8 assay. Relative cell viability (%) was calculated as follows:Viability (%) = (OD_experiment_−OD_blank_)/(OD_control_−OD_blank_) ×100%

### Natural Compound Library

Natural Compound Library was purchased from TargetMol Chemicals Inc (L6000).

### Clinical Analysis of Tissue Microarrays

All sections were scored by two experienced pathologists according to the immunoreactive score (IRS) system.^[^ ^42^ ^]^ Staining intensity was scored as follows: 0 (negative staining), 1 (weak staining), 2 (moderate staining), or 3 (strong staining). The percentage of positive tumor cells was scored as follows: 1 (<10%), 2 (10–35%), 3 (35–70%), and 4 (>70%). The IRS scores were calculated as the product of the staining intensity score and the score of percentage of positive tumor cells.

### Immunohistochemistry (IHC)

When the CDX or PDX tumors reached 100 mm^3^, mice received local RT at 8 Gy. Tumors were isolated at 24 h after IR or at the end point of observation for immunohistochemistry. After tumors were fixed with 4% paraformaldehyde for 24 h, they were embedded in paraffin and cut into 3 µm slices for immunohistochemical analysis of Ki67 (Abcam, #15580), γH2AX (Abcam, #229914), Ku80, Ku70, DNA‐PKcs, USP7 and p‐DNA‐PKcs. Three sections from each sample were examined, and five high‐magnification fields were randomly selected for analysis. The ImageJ software was used to analyze the percentage of positive cells. Staining was automatically scored using the IHC Profiler plug‐in, and positive and negative cells were then counted using the Trainable Weka Segmentation plug‐in. The percentage of positive tumor cells was scored as follows: 1 (<10%), 2 (10–35%), 3 (35–70%), and 4 (>70%). The IRS scores were calculated as the product of the staining intensity score and the score of percentage of positive tumor cells.

### Statistical Analysis

Statistical analysis was performed via two‐tailed unpaired Student's t‐test or two‐way ANOVA. Data was presented as mean ± SD; ^*^ *P* < 0.05, ^**^ *P* < 0.01, ^***^ *P* < 0.001, ^****^ *P* < 0.0001, ns: not significant. All data are representative of three independent experiments. GraphPad Prism 6 (GraphPad Software, La Jolla, CA, USA) was used for all the statistical analyses and graph generation. All the experiments were independently conducted at least three times.

### Ethics Approval and Consent to Participate

The entire study design and protocols were approved by the Animal Protection and Utilization Committee of Hangzhou Institute of Medicine, Chinese Academy of Sciences (Approval No. 2023R0034), in accordance with the Guide for the Care and Use of Laboratory Animals published by the US NIH (Publication No.96‐01). Patient samples and patient‐derived xenograft model experiments were approved by the Ethics Committee of Zhejiang Cancer Hospital (Approval No. IIT‐2025‐247).

### Consent for Publication

All patients who provided human tissue had signed informed consent forms during their hospital stay.

## Conflict of Interest

The authors declare no conflict of interest.

## Author Contributions

The conceptualization of the study was carried out by X.C. and Z.Y. The investigation was conducted by D.Z., C.L., Y.H., H.L., Q.B., M.D., and C.Z. Visualization was performed by Y.H. and G.L. Data curation was undertaken by D.Z., Y.Z., H.H., Y.H., and G.L. Methodology was developed by F.G., J.J., Y.D., and W.W. The original draft was prepared by D.Z. and Y.S., while the review and editing were completed by Z.Y. and S.X. Supervision of the project was provided by X.C. and Z.Y., and funding acquisition was secured by X.C. and Z.Y. All authors have read and agreed to the published version of the manuscript.

## Supporting information



Supporting Information

## Data Availability

The data that support the findings of this study are available in the supplementary material of this article.
