# Supplementary material for: APOL2 Stabilizes Ku80 to Confer NHEJ‐Mediated Radioresistance in Gastric Cancer
Source: Adv Sci (Weinh). 2025 Sep 15;12(44):e06294. doi: 10.1002/advs.202506294 (PMC12667481; doi:10.1002/advs.202506294)
Supplement: Supplementary file 1 — Supporting Information [file ADVS-12-e06294-s001.docx]

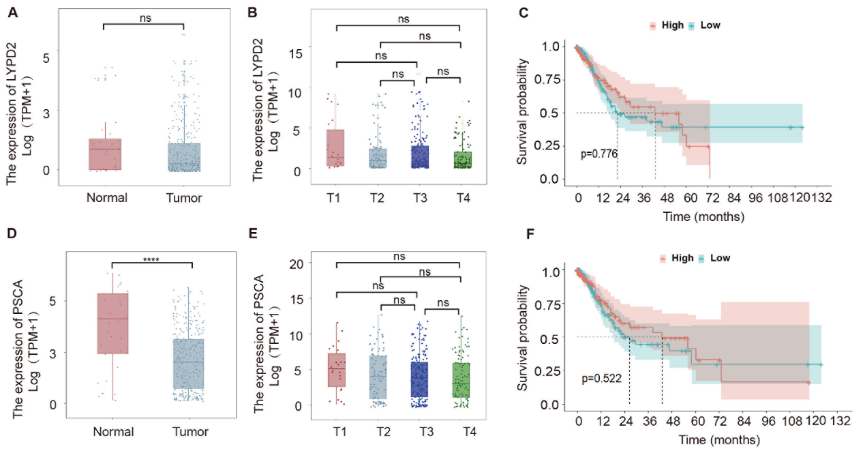


**Supplementary Figure 1** Expression and prognostic correlation of LYPD2 and PSCA in GC patients. A) LYPD2 expression in normal and GC tissues. B) LYPD2 expression across TCGA T stages (T1–T4) in GC. C) KM survival curves comparing OS in GC patients with high versus low LYPD2 expression (log-rank test). (D) PSCA expression in normal and GC tissues. E) PSCA expression across TCGA T stages (T1–T4). F) KM survival curves comparing OS in GC patients with high versus low PSCA expression (log-rank test). Statistical analysis was performed via two-tailed unpaired Student’s t-tests. Data was presented as mean ± SD. ****p<0.0001. All data are obtained from TCGA database.


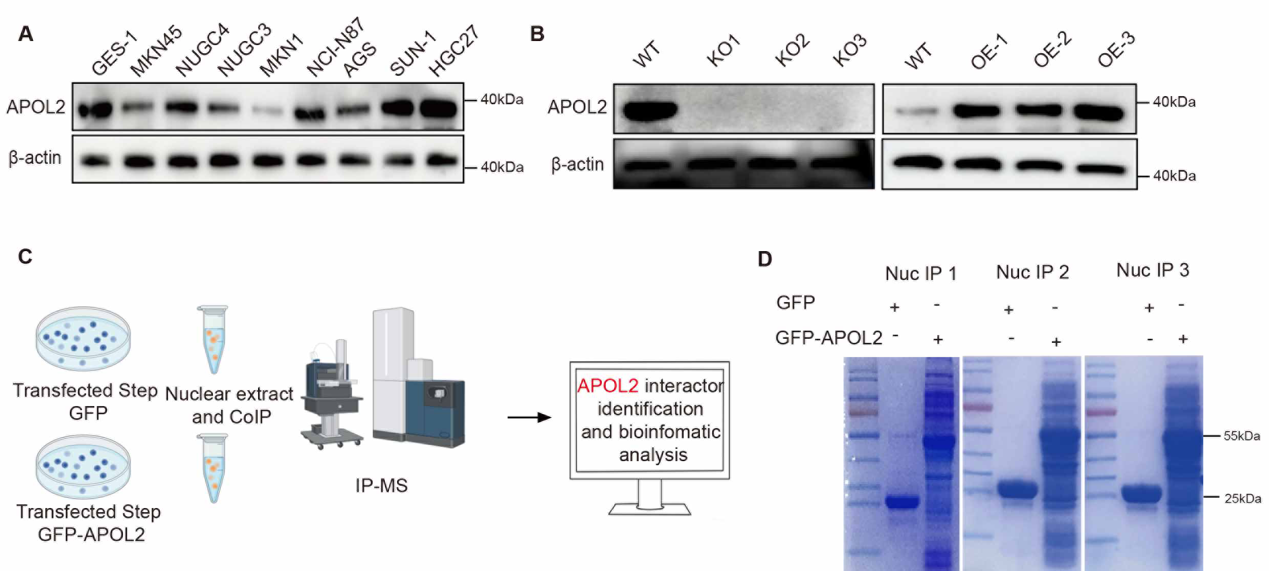


**Supplementary Figure 2** Identification of the nuclear APOL2 interactome. A) APOL2 expression levels across various GC cell lines. B) Validation of APOL2 knockout (KO) and overexpression (OE) stable cell lines by Western blot. HGC27 cells for APOL2 knockout and MKN1 cells were used for APOL2 overexpression. C) Schematic diagram of GFP-APOL2 purification and mass spectrometry. D) Three independent immunoprecipitations (IP 1-3) of nuclear extracts from HEK293T were performed by Coomassie bright blue staining of SDS-PAGE gel for mass spectrometry.


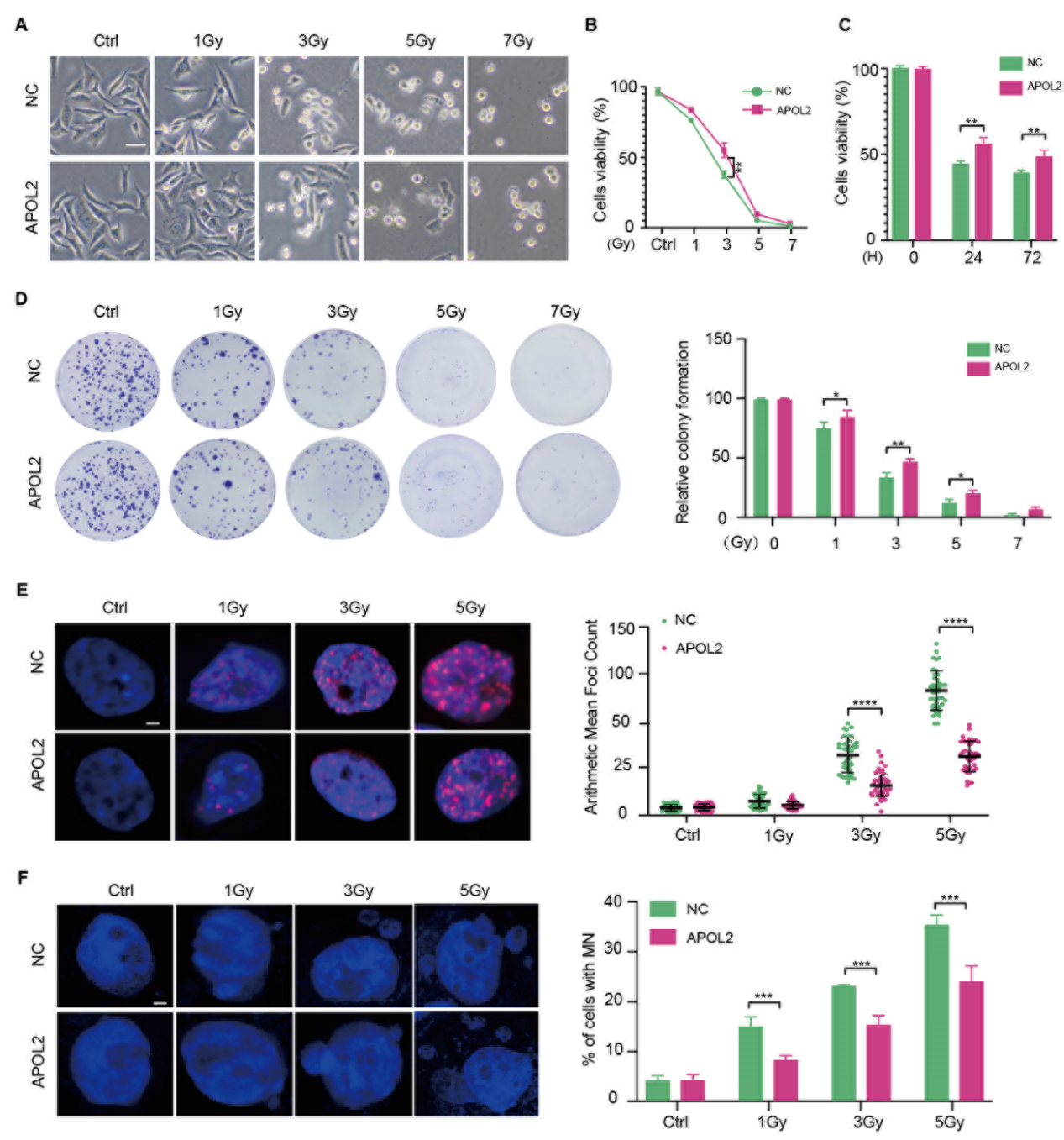


**Supplementary Figure 3** APOL2 promotes radioresistance in GC cells. A) Morphological changes in APOL2-OE cells 24 h after treatment with 0, 1, 3, 5, or 7 Gy IR. Scale bar: 25 μm. B) Cells were irradiated with different doses (1-7 Gy), and cell viability was determined by CCK-8 assay after 24 h. C) Cells were treated with a dose of 3 Gy, and cell viability was detected by CCK-8 assay at 0, 24, and 72 h. D) Representative images and quantitative analysis of colony formation assay under different dose concentrations (1-7 Gy) (n=40). E) Representative immunofluorescence images and quantitative analysis of γ-H2AX foci at different dose concentrations (1-5 Gy) (n=20). Scale bar: 10 μm. F) Representative images and quantitative analysis of micronuclei at different dose concentrations (1-5 Gy). Scale bar: 10 μm. Statistical analysis was performed via two-tailed unpaired Student’s t-tests. Data was presented as mean ± SD; *P<0.05, **P<0.01, ***P<0.001, ****P<0.0001, ns: not significant. All data are representative of three independent experiments.


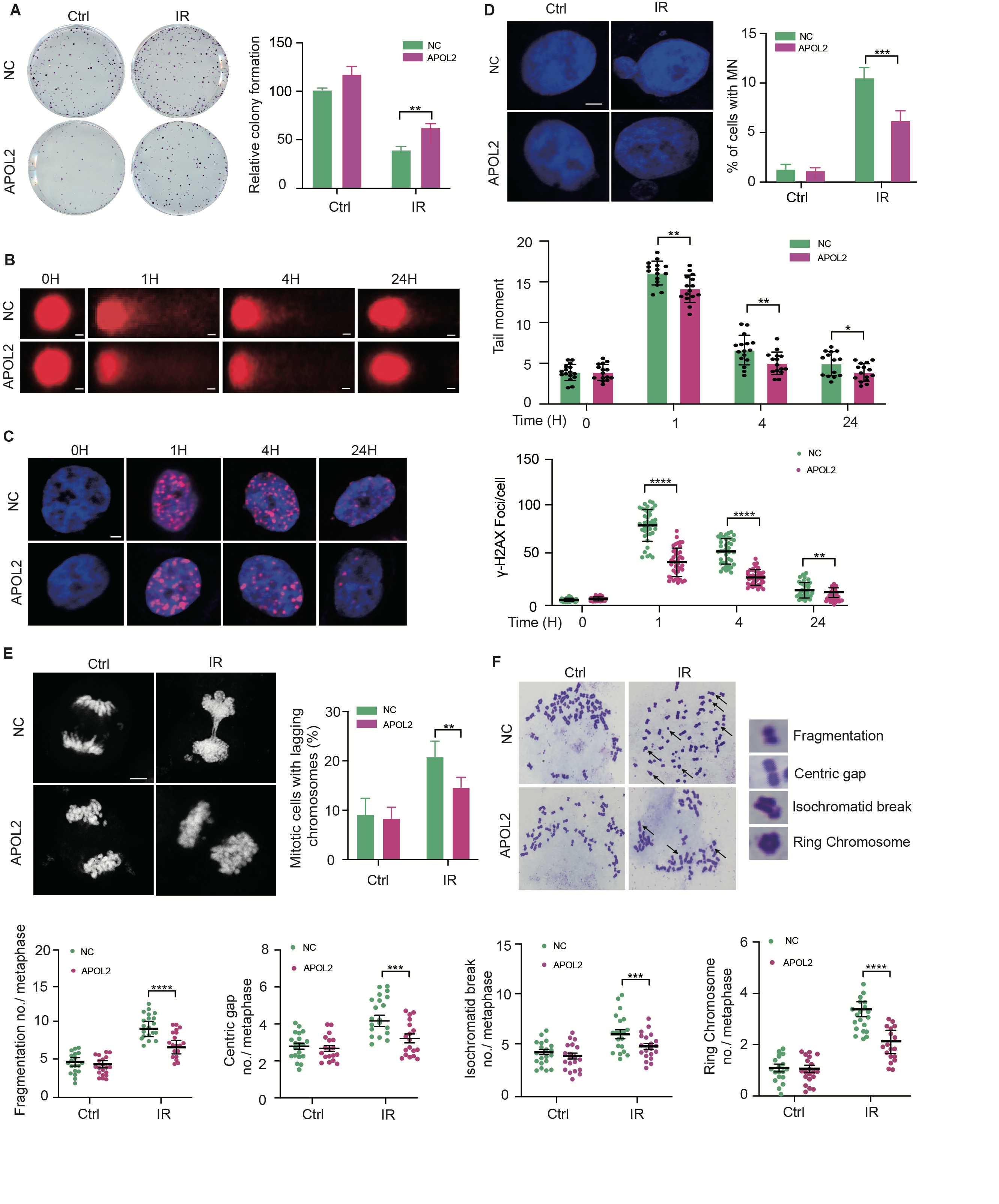


**Supplementary Figure 4** APOL2 enhances DNA repair and genomic stability in GC cells. A) Representative images and quantitative analysis of the colony formation assay in the NC and APOL2 OE groups at a radiation dose of 3 Gy. B) Comet assays were performed to measure tail moments in NC and APOL2-OE cells at indicated time points post-3 Gy IR, reflecting DSB repair capacity (n=15). Scale bar: 10 μm. C) Immunofluorescence analysis of γ-H2AX foci in NC and APOL2-OE cells at 1, 4, and 24 h after 3 Gy IR (n=40). Scale bar: 10 μm. D) Representative images of micronucleus formation 24 h after 3 Gy IR (n=20). DAPI staining highlights nuclei. The percentage of micronucleated cells (MN%) was quantified. Scale bar: 10 μm. E) Representative images and quantification of chromosomal lag in anaphase/telophase cells 24 h after 3 Gy IR (n=20). Scale bar: 25 μm. F) Representative images of chromosomal aberrations and quantitative analysis 24 h after 3 Gy IR in NC and APOL2-OE cells (n=20). Scale bar: 25 μm. Statistical analysis was performed via two-tailed unpaired Student’s t-tests. Data was presented as mean ± SD; *P<0.05, **P<0.01, ***P<0.001, ****P<0.0001. All data are representative of three independent experiments.


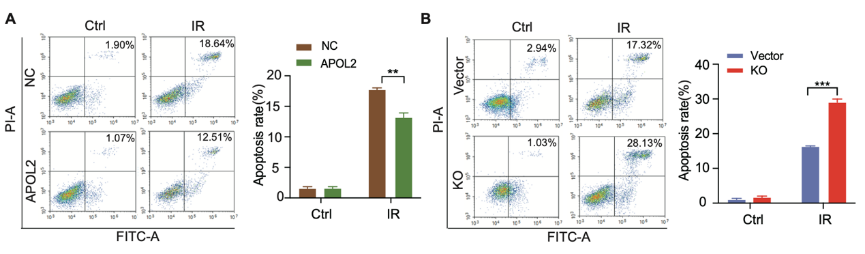


**Supplementary Figure 5** APOL2 inhibits apoptosis in GC cells after irradiation. (A) APOL2-OE reduces apoptosis in MKN1 cells after 3 Gy IR. (B) APOL2-KO increases apoptosis in HGC27 cells after 3 Gy IR. The results were quantified by Annexin V/PI staining and flow cytometry. Statistical analysis was performed via two-way ANOVA. Data was presented as mean ± SD; **P<0.01, ***P<0.001. All data are representative of three independent experiments.


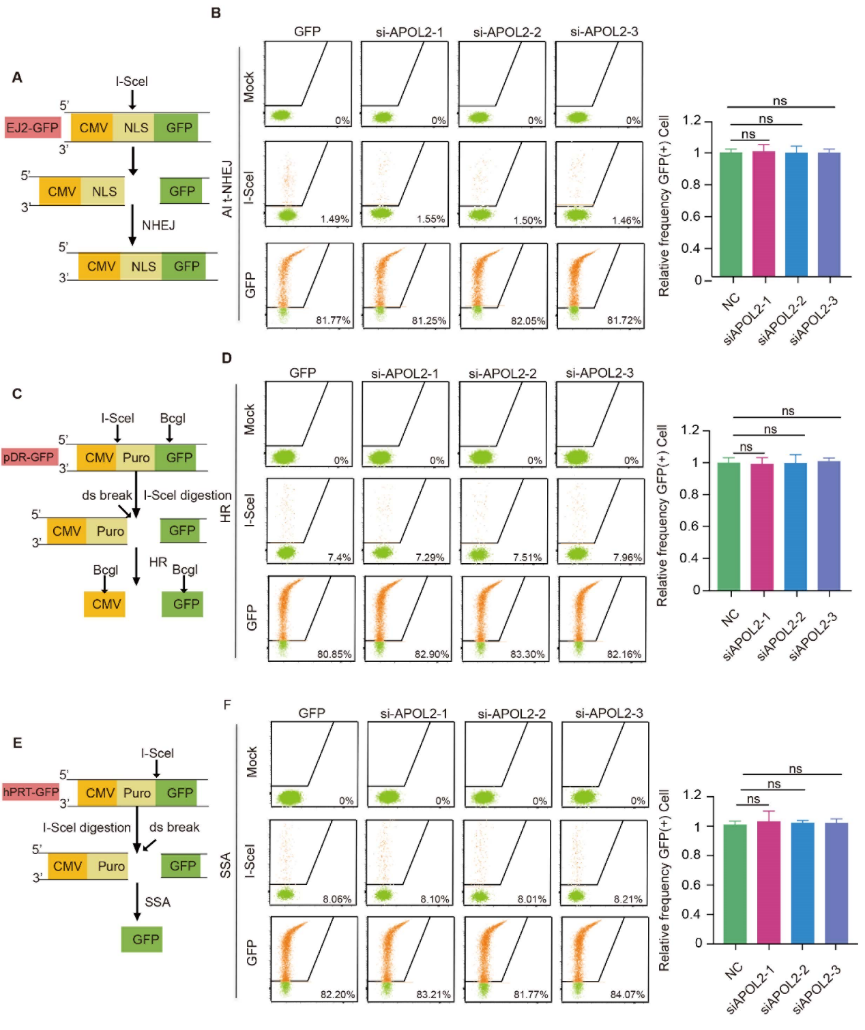


**Supplementary Figure 6** APOL2 regulates the pathway of DNA damage repair in GC. (A, C, E) Schematic diagrams of the reporter plasmids for alt-NHEJ (EJ2-GFP), HR (pDR-GFP), and SSA (hPRT-GFP). Each reporter restores GFP expression upon successful repair of an I-SceI-induced DSB. (B, D, F) Flow cytometry quantification of GFP-positive cells in GC cells co-transfected with I-SceI and each reporter, following APOL2 knockdown (si-APOL2-1/2/3) or NC. Statistical analysis was performed via two-tailed unpaired Student's t-tests. Data was presented as mean ± SD; ns: not significant. All data are representative of three independent experiments.


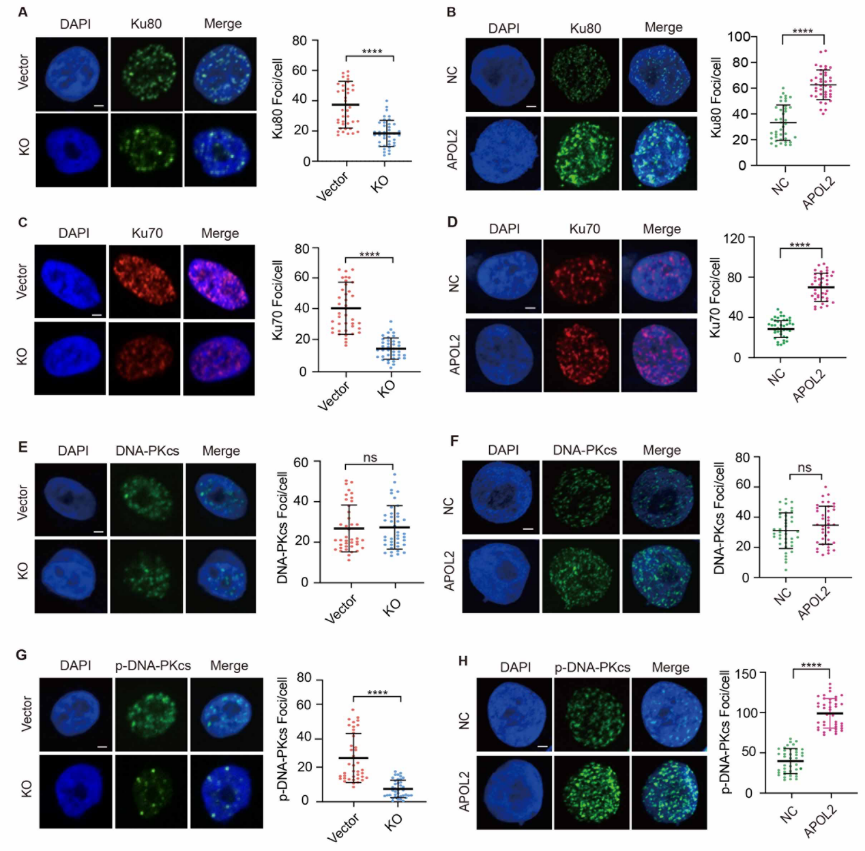


**Supplementary Figure 7** APOL2 enhances the foci formation of NHEJ-related proteins. A-H) Representative immunofluorescence images (left) and quantitative analysis (right) of Ku80/Ku70/DNA-PKcs/p-DNA-PKcs foci formation in A), C), E), G) APOL2-KO cells and B), D), F), H) APOL2-OE cells after 3 Gy irradiation (n=40). Scale, 10 μm. Statistical analysis was performed via two-tailed unpaired Student’s t-tests. Data was presented as mean ± SD; ****p<0.0001, ns: not significant. All data are representative of three independent experiments.


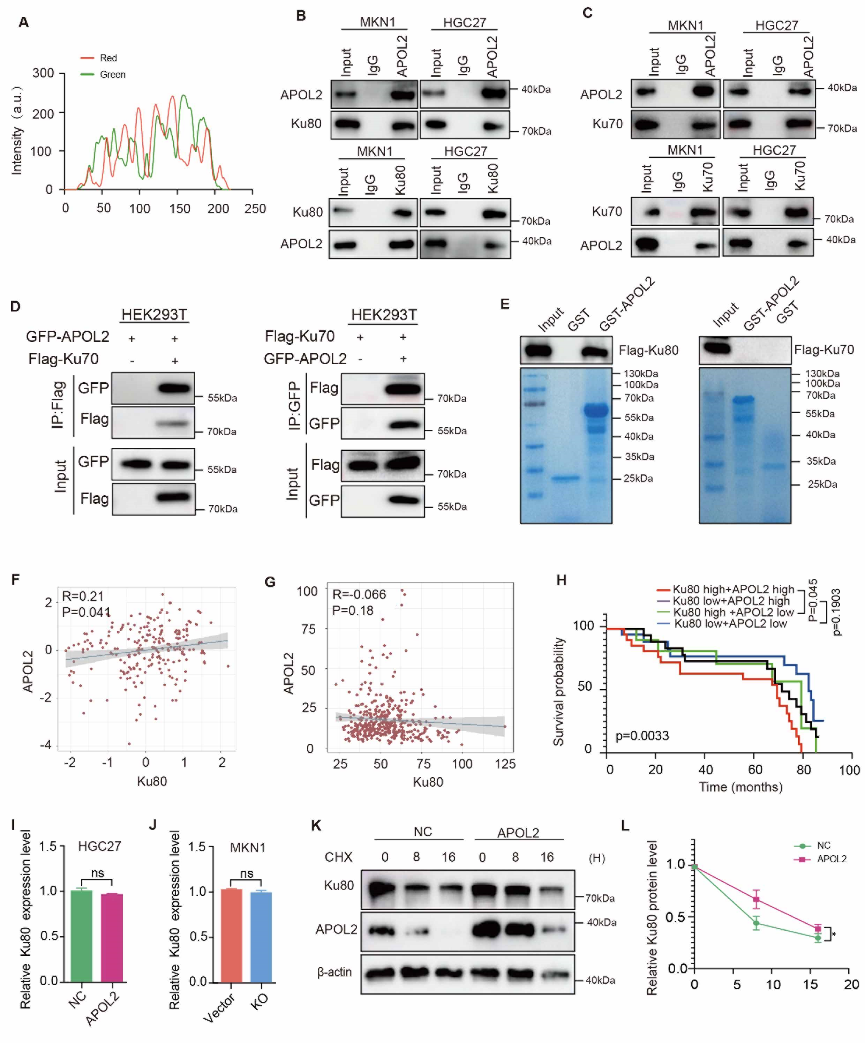


**Supplementary Figure 8** APOL2 binds to Ku80. A) Quantification of co-localization of APOL2 (red) and Ku80 (green) in Figure 3E. B) Endogenous interactions between APOL2 and Ku80 was confirmed in MKN1 and HGC27 cells. Cell lysates were subjected to immunoprecipitation using anti-APOL2 antibody (upper panel) and anti-Ku80 antibody (lower panel), respectively. The precipitated complexes were then detected with anti-APOL2 and anti-Ku80 antibodies. C) Endogenous interaction between APOL2 and Ku70 was verified in MKN1 and HGC27 cells. Immunoprecipitation was performed on cell lysates using anti-APOL2 antibody (upper panel) and anti-Ku70 antibody (lower panel), followed by detection of the precipitated complexes with anti-APOL2 and anti-Ku70 antibodies. D) Exogenous interaction between APOL2 and Ku70 was demonstrated in HEK293T cells co-transfected with GFP-APOL2 and Flag-Ku70 plasmids. Cell lysates were immunoprecipitated with anti-Flag antibody (upper panel) and anti-GFP antibody (lower panel), respectively, and the precipitated complexes were detected using anti-Flag and anti-GFP antibodies. E) In vitro GST pulldown assay results showed that Flag-Ku80 (left) and Flag-Ku70 (right) could directly bind to GST-APOL2, with GST serving as a control. F) Analysis of the relationship between APOL2 and Ku80 at the gene expression level based on proteomic data. G) Bioinformatics analysis revealed the functional association between APOL2 and the Ku80-related gene network. H) KM curve analysis showing the relationship between the expression levels of APOL2 and Ku80 and OS, with grouping based on the expression levels of APOL2 and Ku80 in clinical samples**.** I-J). The expression level of Ku80 mRNA in MKN1 and HGC27 cells were measured by qRT-PCR. K) Assessment of Ku80 protein stability using CHX chase assay. MKN1 cells stably expressing GFP-APOL2 or empty vector (negative control) were treated with CHX (100 μg/mL) for 0, 8, and 16 h. Equal amounts of lysates (quantified by BCA assay) were analyzed by Western blot. L) Statistical graph of Ku80 protein stability in MKN1 cells with empty Vector control (NC) and stable expression of GFP-APOL2. Statistical analysis was performed via two-way ANOVA. Data was presented as mean ± SD; *p<0.05. All data are representative of three independent experiments.


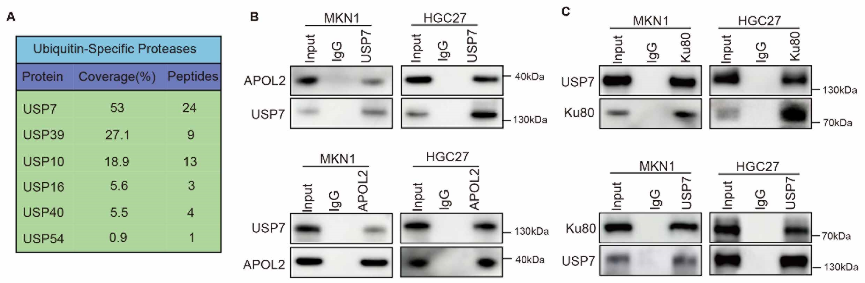


**Supplementary Figure 9** USP7 interacts with APOL2 and Ku80. A) DUB family members identified by LC-MS, with the average coverage and number of unique peptides for each protein listed. B) Endogenous interaction between APOL2 was confirmed in MKN1 and HGC27 cells. Cell lysates were subjected to immunoprecipitation using anti-USP7 antibody (upper panel) and anti-APOL2 antibody (lower panel), respectively. The precipitated complexes were then detected with anti-APOL2 and anti-USP7 antibodies. C) Endogenous interaction between USP7 and Ku80 was verified in MKN1 and HGC27 cells. Immunoprecipitation was performed on cell lysates using anti-Ku80 antibody (upper panel) and anti-USP7 antibody (lower panel), followed by detection of the precipitated complexes with anti-USP7 and anti-Ku80 antibodies. All data are representative of three independent experiments.


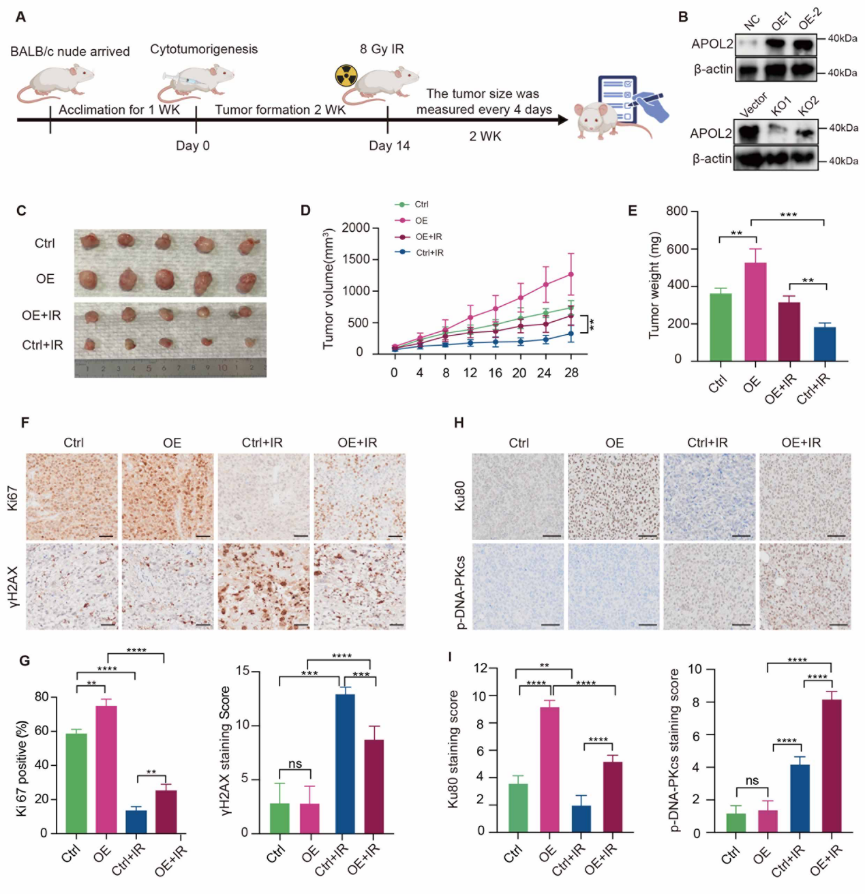


**Supplementary Figure 10** APOL2 suppresses radiosensitivity of GC cells *in vivo*. A) Schematic diagram showing the timeline of animal experiments. B) The overexpression efficiency of APOL2 protein in CDX model and PDX model were detected by Western blot. C) Representative images of different groups of xenograft tumors 28 days after 8 Gy local RT. D-E) Xenograft tumor growth curve (D) and endpoint weight (E) for each xenograft group in the CDX model after 8 Gy local RT (n=5). F-H) Representative IHC staining images of Ki67, γ-H2AX, Ku80 and DNA-PKcs in tumor tissue. F: Scale, 50 μm, H: Scale, 100 μm. G-I) Quantitative analysis of Ki67, γ-H2AX, Ku80 and p-DNA-PKcs by IHC in tumor sections (n=5). Statistical analysis was performed via one-way ANOVA. Data was presented as mean ± SD; **P<0.01, ***P<0.001 and ****P<0.0001. All data are representative of three independent experiments.


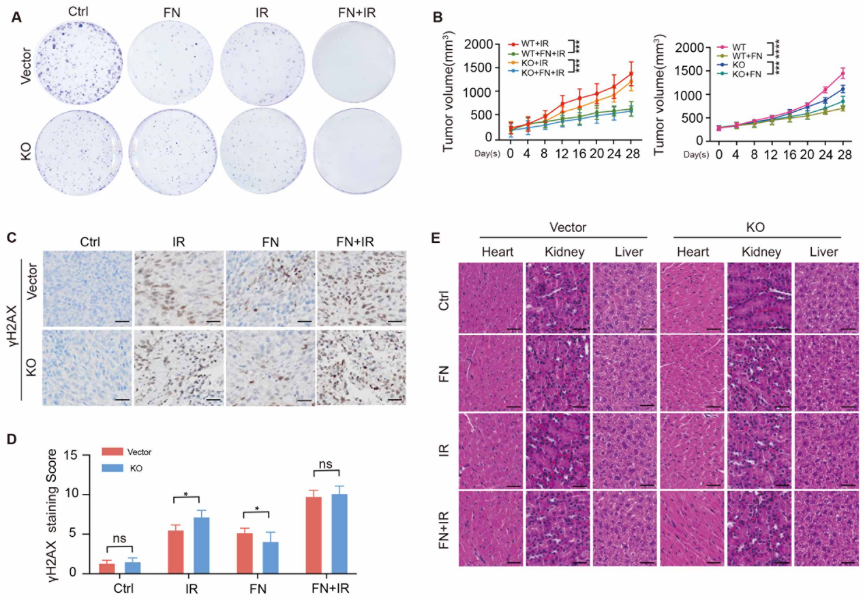


**Supplementary** F**igure 11** A) Colony formation diagram of the vector and APOL2-KO cells after IR RT and FN treatment. B) Statistical graphs of tumor volume growth curves for subcutaneous tumors derived from WT and APOL2-KO cells after treatment with IR RT combined with FN (left) and with FN alone (right, n=5). C) Graphs of immunohistochemical staining for γ-H2AX in tumor tissue after treatment with IR RT combined with FN. Scale bar, 100 μm. D) Statistical graph of γ-H2AX-positive cells in tumor tissues after treatment with IR RT combined with FN (n=5). E) H&E staining of major organ tissues (heart, kidney and liver) from each group of mice (n=5). Scale bar, 100 μm. Statistical analysis was performed via one-way ANOVA. Data was presented as mean ± SD; P<0.05, **P<0.01, ns not significant. All data are representative of three independent experiments.

**Supplementary Table 1 List of primers used in this study.**

| Gene | Sequence (5’ to 3’) | Sequence (3’ to 5’) |
| --- | --- | --- |
| Ku80 | GTTACCTGGAGGCGGATCATCTAATTC | CAAAGGAGGACTCTGGAAAGGGTTAAG |
| siAPOL2-1 | CAGUGUGGUAGAACUAGUAtt | UACUAGUUCUACCACACUGtt |
| siAPOL2-2 | GCGGCACCAAUGUAGCAAAtt | UUUGCUACAUUGGUGCCGCtt |
| siAPOL2-3  siUSP7-1  siUSP7-2  siUSP7-3  siKu80-1  siKu80-2  siKu80-3 | UUUGCUACAUUGGUGCCGCtt  GGAGAAAGCAUCAGGGAAAtt  GGACAUAGACAAAGAGAAUtt  UGAUAAACCUGUAGGAACAtt  GAGAAGAGGCAUAUUGAAAtt  GGACAGUUGUGGAUGCAAAtt  GGAAGAAGCCAUUAAGUUUtt | AUCUUGGUGAGAAAGUUGAGCtt  UUUCCCUGAUGCUUUCUCCtt  AUUCUCUUUGUCUAUGUCCtt  UGUUCCUACAGGUUUAUCAtt  UUUCAAUAUGCCUCUUCUCtt  UUUGCAUCCACAACUGUCCtt  AAACUUAAUGGCUUCUUCCtt |
